# Decoding the genome of *Brainea insignis* reveals insights into fern evolution and conservation

**DOI:** 10.1038/s41467-025-68053-0

**Published:** 2025-12-30

**Authors:** Zengqiang Xia, Lei Duan, Yuhan Fang, Yan Jiang, Hongfeng Chen, Yuehong Yan, Aihua Wang, Zixiang Li, Ziyue Liu, Guohua Zhao, Hui Shen, Yves Van de Peer, Ming Kang, Faguo Wang

**Affiliations:** 1 Guangdong Provincial Key Laboratory of Applied Botany, State Key Laboratory of Plant Diversity and Specialty Crops, South China Botanical Garden, Chinese Academy of Sciences, Guangzhou, China; 2 Eastern China Conservation Centre for Wild Endangered Plant Resources, Shanghai Chenshan Botanical Garden, Shanghai, China; 3 University of Chinese Academy of Sciences, Beijing, China; 4 Key Laboratory of Environment Change and Resources Use in Beibu Gulf, Ministry of Education, and Guangxi Key Laboratory of Earth Surface Processes and Intelligent Simulation, Nanning Normal University, Nanning, China; 5 Shenzhen Key Laboratory of Southern Subtropical Plant Diversity, Fairy Lake Botanical Garden, Shenzhen & Chinese Academy of Sciences, Shenzhen, Guangdong China; 6 Department of Plant Biotechnology and Bioinformatics, Ghent University, Ghent, Belgium; 7 VIB-UGent Center for Plant Systems Biology, Ghent, Belgium; 8 Department of Biochemistry, Genetics and Microbiology, University of Pretoria, Pretoria, South Africa; 9 College of Horticulture, Academy for Advanced Interdisciplinary Studies, Nanjing Agricultural University, Nanjing, China

**Keywords:** Molecular evolution, Population genetics, Comparative genomics, Plant evolution

## Abstract

Ferns are an ancient lineage of vascular plants, yet limited genomic resources constrain both evolutionary and conservation inference. Here, we generate a chromosome-level genome assembly for the endangered cycad fern *Brainea insignis* (8.62 Gb), the sole species in its genus within eupolypods II, and integrate comparative and population genomics to resolve its evolutionary history and vulnerability. The genome retains the ancient whole-genome duplication shared by leptosporangiate ferns; however, its exceptional size is driven primarily by recent repeat accumulation and further shaped by lineage-specific evolutionary signatures linked to functional specialization. Resequencing across the range identifies three geographically and environmentally structured lineages shaped by Quaternary refugia, limited postglacial expansion and localized admixture. Recently reduced populations show pronounced genomic erosion, including inbreeding and elevated genetic load, due to insufficient time for purging. We detect climate-associated local adaptation and project substantial future genetic offsets, with southwestern Indochina populations at highest risk. Our results expand fern genomics and support spatially tailored conservation strategies that maintains habitat connectivity and promotes adaptive gene flow.

## Abstract (web summary)

Limited genomic resources constrain our understanding of fern evolution and conservation. Here, the authors present the genome of *Brainea insignis*, a monotypic endangered fern, and identify evolutionary signatures to guide conservation strategies from comparative and population genomic analyses.

## Introduction

Ferns are among the earliest lineages of vascular plants^1^ and encompass over 13,000 species distributed across diverse ecological niches worldwide^2^. These species exhibit striking morphological and ecological variation—from small, ground-hugging plants to towering tree ferns—and thrive in habitats ranging from tropical rainforests to arid or temperate zones^3,4^. As the sister lineage to seed plants, ferns provide critical insights into macroevolution transitions in plant anatomy, life history, and reproduction^1,5^. Moreover, their high spore production and effective wind dispersal^6^ make many fern species ideal models for investigating gene flow, dispersal patterns, and local adaptation across broad spatial and ecological gradients.

Despite significant progress in seed plant genomics, which has illuminated the complex genomes of many economically and ecologically important species^7–9^, fern genomics remains comparatively understudied. This disparity is largely attributable to the large genome sizes and complex ploidy levels characteristic of many ferns^10,11^. For instance, ferns commonly possess large genomes and intricate chromosome structures, as exemplified by *Tmesipteris oblanceolata*, which has a record-breaking 160-Gb genome^12^. Additionally, most population genetic theories and analytical tools are tailored to diploid species^13,14^, limiting their applicability to ferns, which often deviate from these genetic and genomic norms^10,15^. As a result, fewer than ten fern species currently have fully assembled genomes, leaving large gaps in our understanding of fern diversification, adaptation, and evolutionary trajectories.

Within ferns, eupolypods—subdivided into eupolypods I (Polypodiineae) and eupolypods II (Aspleniineae)—represent roughly two-thirds of extant fern diversity^16^. Despite their ecological and evolutionary significance, no high-quality reference genome has been established for this clade. *Brainea insignis*, commonly known as the cycad fern, belongs to eupolypods II and is the sole species in its genus. This monotypic genus is found in tropical Asia and bears considerable ornamental and medicinal importance^17–19^. It has been listed as a nationally protected species in China since 1999^20^ and identified as a priority protected species in India^21^. In China, *B. insignis* is highly sensitive to environmental disruption and human activities, which have led to precipitous population declines^17^. Given the species’ dual importance to conservation and evolutionary studies, understanding its genomic makeup and evolutionary resilience is critical for guiding the conservation of both the species itself and the biodiverse habitats it sustains^22^.

In this study, we present chromosome-level genome assembly for *B. insignis*, providing an essential genomic resource for the eupolypods II clade. By resequencing 94 individuals from multiple populations, we investigate patterns of genetic diversity, population structure, local adaptation, and the demographic history of this endangered species. Building upon these genomic data, we further estimate genetic offset under future climate scenarios to assess the risks of climate-induced maladaptation. Our results bridge a critical gap in fern genomics, offering fresh insights into the mechanisms underpinning fern genome evolution and informing evidence-based conservation strategies. By addressing key questions regarding the evolutionary potential of *B*. *insignis*, our work not only contributes to the broader understanding of fern diversification but also highlights the importance of genomics-based strategies for preserving biodiversity in a rapidly changing world.

## Results and discussion

### Chromosome-scale genome assembly and annotation

**Fig. 1:**
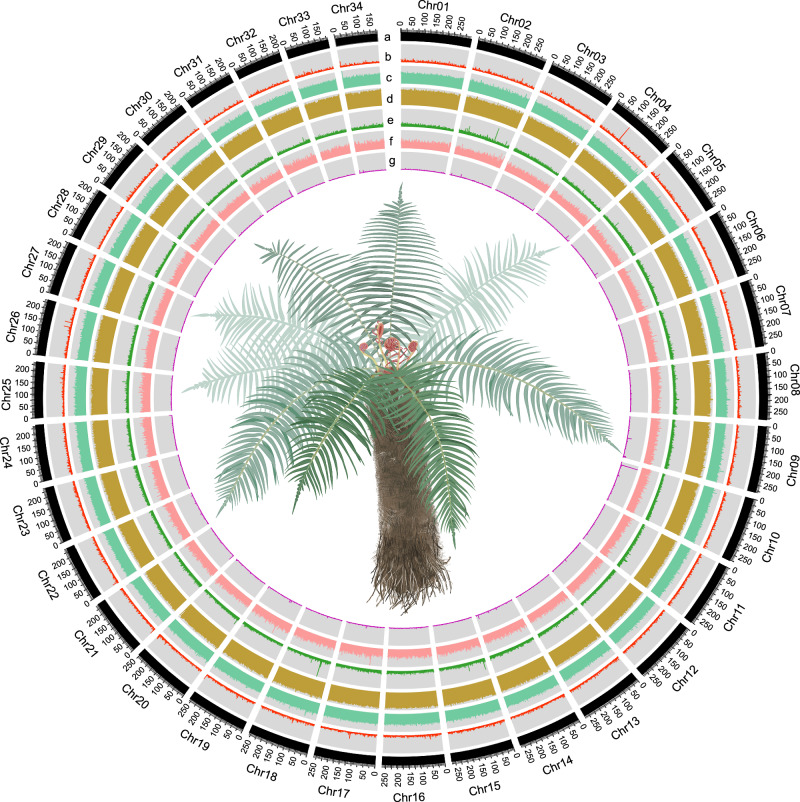
Genome features and morphological illustration of *B. insignis.* The genomic landscape of the 34 *B. insignis* pseudo-chromosomes is shown in non-overlapping 600-kb windows. Panels **a**–**g** correspond to tracks from the outer to inner rings of the circos plot. **a** chromosome length, **b** gene density, **c** GC content, **d** TE coverage, **e** LTR-Copia coverage, **f** LTR-Gypsy coverage, and (**g**) tandem repeats density.

To guide our sequencing strategy, we first determined the genome size and chromosome complement of *B. insignis*. Four independent flow cytometry analyses estimated the genome size at 8.71 Gb (Supplementary Fig. 1), while cytological examination revealed a somatic chromosome number of 2n = 68 (Supplementary Fig. 2). A subsequent genome survey suggested a genome size of approximately 8.40 Gb, with low heterozygosity (0.28%), a high repeat content (89.23%), and diploidy, as indicated by k-mer frequency analyses (Supplementary Fig. 3). Building on these findings, we generated a deep-sequencing dataset to account for the large genome size and the abundance of repeats. We obtained a total of 359.99 Gb (41.76× coverage) of PacBio HiFi reads and 1404.44 Gb (162.92× coverage) of Hi-C reads (Supplementary Table 1 and 2). We then assembled an 8.62 Gb genome, with a contig N50 of 4.36 Mb and a scaffold N50 of 265.61 Mb (Table 1). We anchored 99.81% of the contig length to 34 pseudochromosomes (Fig. 1, Table 1, and Supplementary Fig. 2), corresponding to the 34 chromosomes of the *B. insignis* haploid set (*n* = 34). This represents one of the largest haploid genomes with a chromosome-level assembly reported for a non-seed plant, surpassing many existing seed plant assemblies in size.

**Table 1:** Genome assembly statistics of *B. insignis*

Contig level	Value
Assembly size (Gb)	8.78
Number of contig	5,791
Contig N50 (Mb)	4.36
N50 contig number	626
GC content	41.59%
PE reads mapping rate	99.66%
**Chromosome level**	**Value**
Total length of chromosomes (Gb)	8.62
Scaffold N50 (Mb)	265.41
Anchor ratio	99.81%
BUSCO completeness	97.4%
LAI	10.34
R-AQI/S-AQI	96.17/98.73

LAI: LTR assembly index; R-AQI: regional assembly quality; S-AQI: structural assembly quality.

We assessed the quality of the assembled genome using multiple approaches. First, 99.66% of Illumina short reads (excluding supplemental and secondary reads) mapped to the assembly. Second, BUSCO analysis (viridiplantae_odb12 dataset; updated July 1, 2025) indicated that 97.4% of the 822 conserved genes were completely recovered (Supplementary Table 3). Third, the assembly achieved a LTR Assembly Index (LAI) score of 10.34, surpassing the reference-level threshold of 10. Finally, Clipping Information for Revealing Assembly Quality (CRAQ) analysis showed strong structural accuracy, with a regional assembly quality (R-AQI) of 96.17 and a structural assembly quality (S-AQI) of 98.73. These results collectively underscore the high contiguity, consistency, and completeness of our *B*. *insignis* assembly.

Repetitive elements, particularly transposable elements (TEs), account for a large fraction of fern genomes^23,24^, and *B. insignis* is no exception. Overall, 7.06 Gb (81.77%) of the genome comprises repeat sequences (Supplementary Table 4), aligning with the genome survey estimate of 89.23% repetition (Supplementary Fig. 3). Long terminal repeat retrotransposons (LTR-RTs) dominate this repeat landscape, accounting for 47.99% of the genome, followed by DNA transposons (DNATs) at 24.49%. The remainder includes non-LTR retrotransposons (e.g., LINEs and SINEs) and a small fraction (2.8%) of unclassified repeats (Supplementary Table 4).

We predicted a total of 43,573 protein-coding genes within the assembled genome (Supplementary Fig. 4a; Supplementary Table 5), with 89.78% of these genes having functional annotations in six major databases (Supplementary Fig. 4b and Supplementary Table 6). The structural characteristics of these genes are broadly consistent with those reported for other fern species (Supplementary Fig. 5): on average, each gene is 15,096.24 bp in length, contains 3.84 exons with a mean exon length of 282.76 bp, and has introns spanning 4,939.39 bp (Supplementary Table 5). Additional BUSCO analysis showed that 692 (84.2% of 822) viridiplantae_odb12 genes were present as complete genes in the annotation (Supplementary Table 3). Collectively, these metrics illustrate the robust quality of our structural and functional gene predictions for *B. insignis*.

### Genomic structural features and comparative genomics

We first examined the structural attributes of the *B. insignis* genome by profiling gene density, GC content, and the distribution of transposable elements (TEs), particularly long terminal repeat retrotransposons (LTR-RTs) from the Gypsy and Copia families (Fig. 1). Both genes and repeats showed a relatively uniform distribution across the genome, in contrast to the more localized patterns typically observed in seed plants (e.g., *Glycine max* and *Miscanthus floridulus*), where gene density generally increases near chromosome termini^25,26^. Similar homogeneity in genomic architecture has been reported for other homosporous fern genomes, including *Ceratopteris richardii*^27^, *Alsophila spinulosa*^28^, and *Adiantum capillus-veneris*^29^, as well as in select lycophytes (*Isoetes taiwanensis*^30^, *Huperzia asiatica*^31^, *Diphasiastrum complanatum*^31^). Although ferns and seed plants share a common ancestor, these findings suggest that lycophytes and ferns may exhibit more similar genomic structural patterns than seed plants. Accordingly, the differences in gene density and repeat coverage between seed-free and seed-bearing vascular plants may be more nuanced than previously assumed^24,29^.

**Fig. 2:**
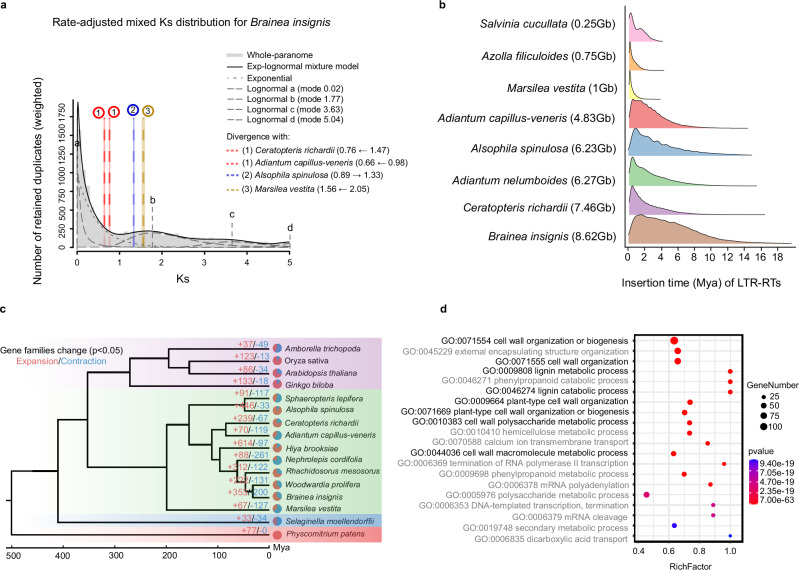
Comparative genomics analyses. **a** Rate-adjusted mixed *K*_s_ distribution of *B*. *insignis*. The whole paranome *K*_s_ distributions are overlaid with rate-adjusted divergence events (speciation events) in colored vertical lines and boxes. As described in Chen et al.^80^, the overall mixture model, represented as the dark solid KDE curve, is made up of an exponential component (dotted grey curve) and optimized log-normal components (dashed grey curves). The log-normal components are labelled with letters. The horizontal arrows indicate the *K*_s_ shifts resulting from substitution rate adjustment. **b** Estimated LTR-RT insertion time distributions for ferns, calculated with lineage-specific substitution rates. **c** Significant gene family expansion and contraction among 16 plant species, including one bryophyte (outgroup), one lycophyte, four seed plants, and ten ferns. Red and blue numbers above branches denote expansion and contraction events, respectively. Statistical significance was assessed using CAFE’s birth–death model (two-sided), and *P* values were obtained directly from CAFE without additional multiple-comparison correction. **d** GO enrichment of the 353 significantly expanded gene families in *B*. *insignis*. Enrichment was tested using a one-sided hypergeometric test, and *P* values were corrected using the Benjamini–Hochberg FDR method (significant at corrected *P* ≤ 0.05). RichFactor denotes the ratio of expanded to total annotated genes per GO term; bubble size shows gene number and colour indicates the adjusted *P* value.

Whole-genome duplication (WGD) is a pivotal force shaping genome architecture and driving evolutionary innovation^15^. Synonymous substitution rates (*K*_s_) distributions are constructed by calculating the *K*_s_ between pairs of homologous genes. To explore the role of WGD in *B. insignis*, we employed a rate-corrected *K*_s_ distribution^32^, which allowed the rescaled orthologous divergence times to be comparable with the paranome *K*_s_ distribution of the focal species. A prominent *K*_s_ peak at 1.77 suggests an ancient WGD event predating the divergence of *Ceratopteris richardii, Adiantum capillus-veneris, Alsophila spinulosa*, and *Marsilea vestita* (Fig. 2a). Further analysis of paralogous *K*_s_ distributions and collinearity corroborated this ancient WGD^33^, pinpointing a consistent peak ( ~ 1.7) and revealing no subsequent, lineage-specific WGDs (Supplementary Figs. 6 and 7). These findings align with genome analyses of *A. capillus-veneris*, which also exhibit only this ancient WGD shared among core leptosporangiate ferns^29^.

Interestingly, the *K*_s_ peak value for *B*. *insignis* (~1.7) is lower than that of *A*. *capillus-veneris* (~2.15), implying a comparatively slower synonymous substitution rate in *B*. *insignis*. To investigate this further, we quantified synonymous (dS) and nonsynonymous (dN) substitution rates in 16 fern species. *B*. *insignis* and its close relatives (*Woodwardia prolifera*) showed significantly lower dS and dN values than many other ferns (Supplementary Fig. 8). Indeed, relative rate tests indicate that *B*. *insignis* exhibits a significantly slower evolutionary rate compared to most other core leptosporangiate ferns (Supplementary Table 7), even slower than certain tree fern species (e.g., *Alsophila spinulosa*). Whole-genome collinearity with the tree fern *A*. *spinulosa* revealed extensive one-to-one syntenic blocks in *B*. *insignis* (Supplementary Fig. 9). Despite over 200 million years of divergence (TimeTree5), the breadth of this conserved collinearity indicates that the *B*. *insignis* genome has evolved comparatively slowly, at least in terms of genomic architecture. Although *B*. *insignis* exhibits a higher ω (dN/dS) ratio than most core leptosporangiate ferns (Supplementary Fig. 10), this may reflect relaxed selective pressures—possibly owing to a stable habitat or small effective population size—rather than accelerated protein evolution. In turn, this slow evolutionary rate may have bolstered genomic stability, enabling *B. insignis* to persist as the sole species in its genus, although it could also limit its adaptive potential in the face of rapid environmental change.

In addition to WGD, variations in LTR-RT content strongly influence genome size evolution in many plant lineages^24,34,35^. However, their specific impact on fern genome expansion remains poorly understood, largely due to limited genomic data. To address this gap, we compared LTR-RT composition and insertion times in eight fern genomes. Our findings indicate that larger fern genomes tend to harbor a higher proportion of LTR-RTs with earlier insertion time (Fig. 2b; Supplementary Fig. 11). While these observations suggest a positive correlation between LTR-RT activity and genome size expansion in ferns, the limited number of available fern genomes hampers broad statistical inferences. Consequently, more extensive sampling and high-quality assemblies are needed to better understand how LTR-RT dynamics have shaped the remarkably diverse genome architectures across ferns.

To further investigate gene family evolution in the *B. insignis* genome, we constructed a high-confidence phylogeny for 16 species using 103 single-copy gene families (Fig. 2c). We identified 353 significantly expanded and 200 significantly contracted gene families on the branch leading to *B. insignis*, with the expanded families enriched in biological processes related to cell wall organization and lignin metabolism (“plant-type cell wall organization”, “lignin metabolic process” and “lignin catabolic process”; Fig. 2d). Gene families involved in monolignol biosynthetic pathway exhibit *K*_a_/*K*_s_ < 1 in *B. insignis* and two other tree ferns (Supplementary Fig. 12), and micro-synteny analyses further reveal that a subset of these lignin-related genes is highly conserved across species (Supplementary Fig. 13). These findings suggest that the ancestral lineage of *B. insignis* evolved specialized lignin-related traits—such as robust, lignified structures—that have been retained in the modern species, while key metabolic pathways remained long-term functional stability. Interestingly, a recent study showed that *Stenochlaena palustris*, a member of the same family, possesses remarkable lignin architectures^36^, indicating that S-lignin production evolved independently in ferns. Although lignin composition in *B*. *insignis* remains to be characterized, the observed expansion and synteny suggest lineage-specific elaboration of lignin-related capacities. Together, these observations are consistent with lineage-specific diversification of lignin pathways in ferns.

Overall, our findings demonstrated that an ancient WGD, repetitive-element dynamics, and gene family expansions have collectively sculpted the genome of *B. insignis*. Although *B. insignis* shares a deep polyploidization event with other core leptosporangiate ferns, it exhibits a slower evolutionary rate and a larger genome, underscoring the complexity of diploidization processes and the importance of repetitive elements in shaping fern genome diversity.

### Population structure and demographic history

**Fig. 3:**
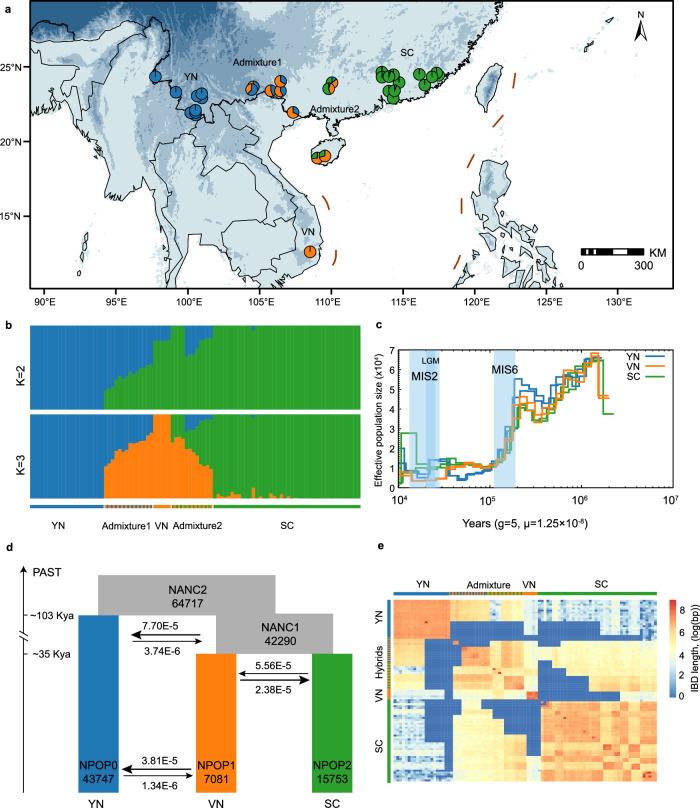
Genetic structure and demographic history of *B*. *insignis.* **a** Map showing sampling locations and overall genetic structure. **b** Population structure of 94 individuals based on ADMIXTURE analysis at *K* = 2 and 3. **c** Demographic history of the three lineages of *B. insignis*, with the three glacial events (MIS2, LGM, and MIS6) highlighted in light blue. **d** Schematic of the best demographic scenario inferred with fastsimcoal2. Numbers at the bottom indicate the estimated *Ne* of each lineage; and the bidirectional arrow shows the direction of gene flow. **e** Heatmap illustrating haplotype sharing among individuals, where colors represent the total length of identity-by-descent (IBD) blocks for each pairwise comparison.

We resequenced 94 *B. insignis* individuals from 29 geographic locations to an average depth of 21.81×, generating 17.79 Tb of raw data (Fig. 3a; and Supplementary Fig. 14 and Supplementary Data 1). After mapping paired-end reads to the *B. insignis* reference genome and applying stringent filtering criteria, we identified 75,060,153 high-quality SNPs and 9,414,109 core variants defined as a high-confidence SNPs set retained after stringent quality control (Supplementary Fig. 15). Dataset 1-3 were derived from these variants and showed no evidence of chromosomal bias (Supplementary Fig. 16).

To elucidate the genetic structure of *B. insignis*, we applied several methods, including ADMIXTURE, principal components analysis (PCA), neighbor-joining (NJ) trees, and chloroplast haplotype networks. ADMIXTURE identified two primary clusters (K = 2), differentiating Yunnan populations (YN lineage) from those in southern China (SC lineage). The optimal number of clusters was determined to be three (K = 3), revealing a distinct lineage from Vietnam (VN lineage) and two apparent admixture zones (Admixture1 and Admixture2) (Fig. 3b; and Supplementary Fig. 17). Additional analyses corroborated this tri-lineage pattern: (1) the chloroplast haplotype network revealed clear distinctions among the YN, VN, and SC lineages, with apparent admixture haplotypes (Supplementary Fig. 18); and (2) PCA and NJ trees confirmed genetic separations corresponding to these three main lineages while highlighting intermediate signatures in the admixture zones (Supplementary Fig. 19). Together, these results show strong isolation by distance (IBD) and isolation by environment (IBE), suggesting that both geographic distance and environmental heterogeneity jointly influence the spatial distribution of genetic variation. This pattern is consistent with multiple glacial refugia during the Quaternary, followed by post-glacial expansions and secondary contact among lineages.

We reconstructed the demographic trajectories of each lineage using the pairwise sequentially Markovian coalescent (PSMC) method (Fig. 3c). All lineages exhibited similar initial trends: an increase in effective population size (*N*e) peaking around 2 million years ago (Mya), followed by a protracted decline until approximately 300 thousand years ago (Kya). A subsequent expansion culminated in a sharp bottleneck around 200 Kya, coinciding with the onset of Marine Isotope Stage 6 (MIS6) glaciation^37,38^. These observations mirror the glacial-contraction and interglacial-expansion cycles reported for other East Asian relict species, including *Ginkgo biloba*^39^, *Cercidiphyllum japonicum*^40^, and *Davidia involucrata*^41^. After the MIS6 bottleneck, the YN lineage diverged from the common ancestor around 100 Kya, whereas the VN and SC lineages separated around 40 Kya. Each lineage subsequently maintained relatively small and stable *Ne* values up to the present (Fig. 3c; and Supplementary Fig. 20).

To complement the PSMC analyses, we performed coalescent-based simulations for each lineage using fastsimcoal2 and identified the most likely speciation model among five initial scenarios without introgression (Supplementary Fig. 21 and 22; Supplementary Table 8 and 9). After determining the best-fitting model, we tested nine additional models incorporating gene flow (Supplementary Fig. 23; and Supplementary Table 10). The optimal model corroborated the PSMC-derived divergence times (Fig. 3d) and provided a robust framework for understanding the evolutionary history of *B. insignis*, further supporting its status as a relict fern species^17,20^. Our simulations also revealed variations in gene flow across different divergence phases. Substantial sharing of identical-by-descent haplotypes between the YN/SC lineages and the admixture zones (Admixture1 and Admixture2) implies relatively recent inter-lineage gene flow (Fig. 3e). Furthermore, TreeMix analysis, with an optimal migration edge of 1, indicates recent gene flow from YN to VN (Supplementary Fig. 24), underscoring ongoing genetic exchange and secondary contact among geographically proximate populations.

### Genomic effects of recent population decline on inbreeding and genetic load

**Fig. 4:**
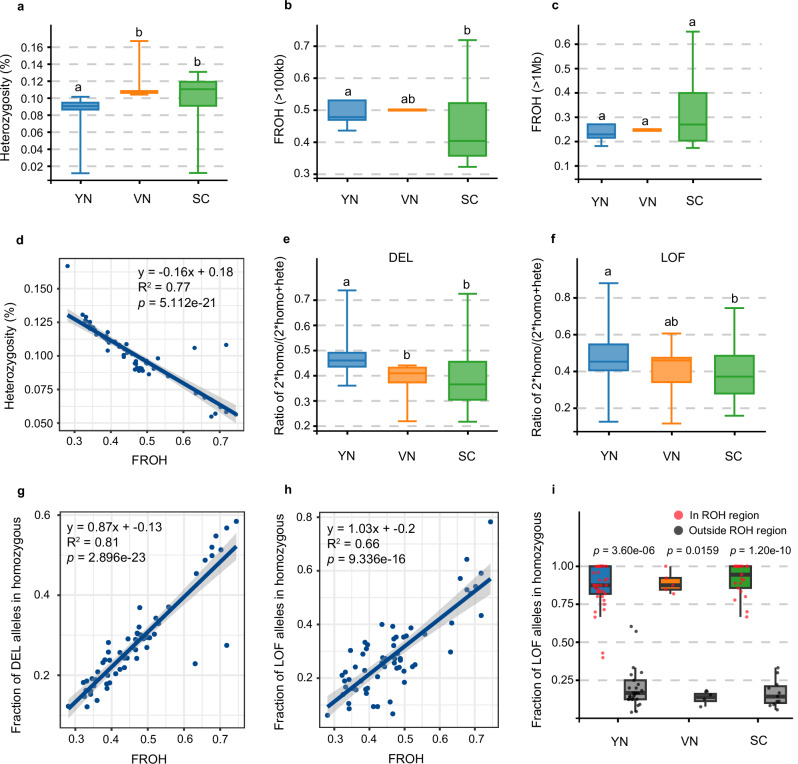
Characterization of inbreeding and genetic loads in *B*. *insignis.* Population genetic characteristics of three lineages (YN: *n* = 21, VN: *n* = 5, SC: *n* = 42 individuals). For all box plots, the box represents the 25th–75th percentiles, the center line indicates the median, and whiskers show the minimum and maximum values. Different lowercase letters (**a**, **b**) indicate significant differences between lineages. **a** Genome-wide heterozygosity estimates of three lineages. Exact *P* values: YN vs. VN = 0.00003, YN vs. SC = 0.00063, VN vs. SC = 0.77612. **b** Inbreeding levels (*F*_ROH_) based on medium-length segments ( > 100 kb). Exact *P* values: YN vs. VN = 0.85, YN vs. SC = 0.014, VN vs. SC = 0.7. **c** Inbreeding levels (*F*_ROH_) based on long segments ( > 1 Mb). Exact *P* values: YN vs. VN = 0.71, YN vs. SC = 0.45, VN vs. SC = 0.27. **d** Regression of genome-wide heterozygosity against *F*_ROH_. **e** Genetic load measur**e**d by the ratio of homozygous DEL (2*homo) to the sum of homozygous and heterozygous deleterious (homo + hete). Exact *P* values: YN vs. VN = 0.0152, YN vs. SC = 0.0015, VN vs. SC = 0.9854. **f** Genetic load measured by the ratio of homozygous LOF (2*homo) to the sum of homozygous and heterozygous LOF (homo + hete). Exact *P* values: YN vs. VN = 0.52, YN vs. SC = 0.04, VN vs. SC = 0.72. **g** Regression of the fraction of homozygous DEL alleles in against *F*_ROH_. **h** Regression of the fraction of homozygous LOF alleles against *F*_ROH_. **i** Comparison of homozygous LOF variant sites within ROH regions vs. outside ROH regions. The dot box plot shows the number of homozygous LOF variants in ROH and non-ROH regions. Statistical notes: For **a**–**c**, **e**, **f**, and **i** significant differences between lineages were assessed using two-sided Wilcoxon rank-sum tests. For the regression analyses (**d**, **g**, and **h**), the significance of the regression coefficients was evaluated using two-sided tests, and the shaded areas represent the 95% confidence intervals (CIs) of the regressions. Source data are provided as a Source Data.

Although effects of population declines are well-documented in many seed plants and animal lineages, the genomic consequences of population decline in ferns remain largely unexplored^42^. To address this gap, we analyzed genetic diversity, inbreeding, and genetic load in *B. insignis*. We hypothesized that the consistently low effective population size (*N*e) since the last bottleneck, compounded by severe declines in recent decades, has led to further losses of genetic diversity. Indeed, genome-wide heterozygosity in the three lineages (YN, VN, and SC) remains low, averaging 0.08, 0.12, and 0.10, respectively (Fig. 4a). Accordingly, the YN lineage exhibits the lowest nucleotide diversity (mean π = 1.043 × 10^−3^), followed by VN (1.126 × 10^−3^) and SC (1.379 × 10^−3^) (Supplementary Fig. 25). YN also exhibits slower linkage disequilibrium (LD) decay, which is consistent with its lowest level of genetic diversity. Genetic divergence (*F*_ST_) values (0.116–0.320) correlate with the geographic distances between the lineages, while positive Tajima’s *D* values corroborate the demographic bottlenecks inferred from our historical reconstructions.

To quantify inbreeding, we identified runs of homozygosity (ROH) in each lineage. All three lineages show numerous long ROHs (>100 kb), with the fraction of the genome in ROH (*F*_ROH_) averaging 0.51 (YN), 0.50 (VN), and 0.43 (SC) (Fig. 4b). Furthermore, a substantial proportion of ROHs exceed 1 Mb (Fig. 4c), indicating pronounced inbreeding. We observed a strong negative correlation between individual heterozygosity and *F*_ROH_ (*R*^2^ = 0.77, *P* < 0.001), confirming that high levels of inbreeding are reducing genome-wide diversity (Fig. 4d).

Next, we assessed genetic load by examining allelic states at loss-of-function (LOF) and missense (deleterious) variants. The ratio of nonsynonymous to synonymous (π_0_/π_4_) diversity was relatively high, ranging from 0.431 to 0.503 (Supplementary Table 11), suggesting that purifying selection on 0-fold sites has been weak or relaxed in *B. insignis*. To put our estimates in a broader context, we conducted a comparative analysis of π₀/π₄ ratios across more than 40 plant species, spanning ferns and seed plants (Supplementary Fig. 26). The endangered ferns (*B. insignis* and *Alsophila* spp.) exhibit markedly elevated π₀/π₄ ratios relative to most seed plants, with the notable exception of *Acer yangbiense*, a species characterized by an extremely small population size. These results support the expectation that small population sizes weaken purifying selection and inflate genetic load across disparate plant lineages. Although absolute values may vary with sampling and analytical pipelines, the qualitative pattern is robust. Deleterious (DEL) and LOF mutations in the homozygous state—an indicator of genetic load^43,44^—are most frequent in the YN lineage (Fig. 4e, f). Moreover, applying a Grantham Score threshold of 150 further underscores the elevated fraction of deleterious missense variants in YN lineage (Supplementary Fig. 27). The frequency of homozygous DEL and LOF alleles correlates with *F*_ROH_ (Fig. 4g, h). We also observed a higher density of LOF variants within ROH segments than outside them (Fig. 4i), highlighting the role of inbreeding in exacerbating genetic load. Functional annotations link LOF-afflicted genes to stress resistance and DNA repair (Supplementary Data 2)—processes vital for organismal survival—implying that accumulated deleterious mutations may diminish population fitness.

Collectively, our findings demonstrate that *B. insignis* harbors extremely low genetic diversity in all three lineages and exhibits pronounced inbreeding and genetic load, particularly in the YN lineage. This pattern contrasts with long-term small populations (e.g., *Alsophila spinulosa*)^42^, where signals of purging have been reported^43,45–48^. In *B. insignis*, however, a recent and severe population contraction likely limited purging opportunities, thereby facilitating the accumulation of deleterious alleles. Consequently, these results underscore that fern lineages shaped by different demographic histories can display sharply contrasting patterns of genetic load purging. In the case of *B. insignis*, the elevated genetic load presents serious challenges to its long-term viability, emphasizing the need for conservation measures that safeguard both habitat protection and the genetic health of the remaining populations.

### Adaptive differentiation and genetic vulnerability under future climate change

**Fig. 5:**
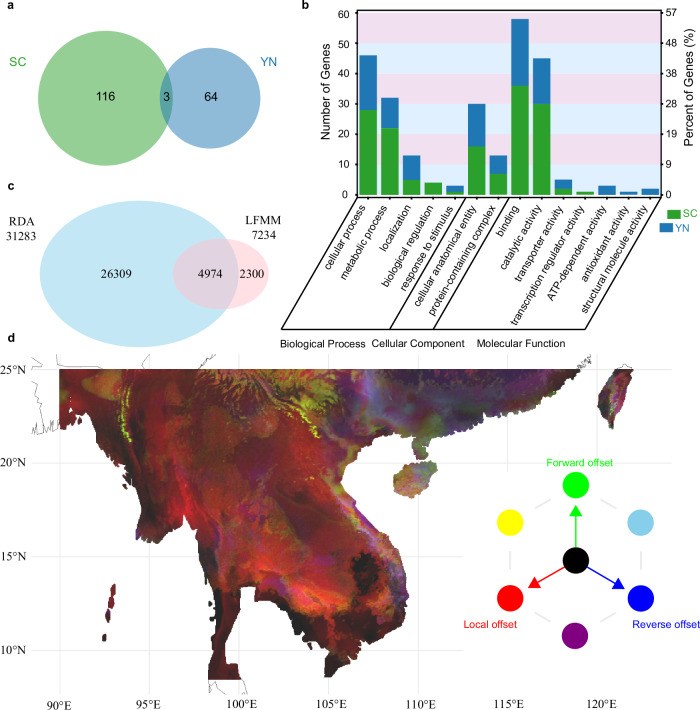
Adaptive differentiation and genetic incompatibility analyses. **a** Venn diagram of selectively swept genes identified in the two lineages. **b** GO annotation of the selected genes in each lineage. **c** Venn diagram showing overlapping loci identified by LFMM and RDA analyses. **d** RGB map of genetic offsets across the *B*. *insignis* range for 2081-2100 under the SSP585 scenario. Brighter cells indicate higher values for each of the three axes, whereas darker cells indicate lower values.

To identify genes potentially involved in lineage-specific adaptations, we conducted selective sweep analyses on the southern China (SC) and Yunnan (YN) lineages, which had large sample sizes and exhibited the highest genetic differentiation. We detected 3,846 positively selected sites (involving 116 genes) in the SC lineage and 2,299 such sites (encompassing 64 genes) in the YN lineage (Fig. 5a; and Supplementary Fig. 28). Only three of these genes were shared between the two lineages. Gene Ontology (GO) analysis revealed that most positively selected genes were linked to core cellular and metabolic processes, including binding and catalytic functions (Fig. 5b). Although the SC and YN lineages shared relatively few positively selected genes, they exhibited considerable overlap in functional categories, suggesting functional convergence on likely adaptive traits. Nonetheless, differences in gene counts per category and lineage-specific annotations point to diverging selection pressures that have driven adaptive differentiation.

Next, we examined the relationship between allele frequency and environmental variables in the context of local adaptation. We identified 4,974 core adaptive variants (Fig. 5c) that displayed significant isolation-by-distance (Mantel’s *r* = 0.4301, *P* < 0.05) and isolation-by-environment (Mantel’s *r* = 0.4058, *P* < 0.05) patterns (Supplementary Fig. 19). However, after controlling for geographic and environmental effects separately, neither relationship remained significant (partial Mantel tests: *P* = 0.147 for IBE, *P* = 0.179 for IBD), suggesting that both factors jointly shape adaptive genetic variation. Redundancy analysis (RDA) identified three distinct genetic clusters corresponding to the YN, SC, and VN lineages, with admixture populations occupying intermediate positions (Supplementary Fig. 29). The YN lineage correlated with a high diurnal temperature range (bio2), while the SC lineage was linked to low bio2, high annual mean temperature (bio1), and high precipitation in the driest month (bio14). In contrast, the VN lineage formed its own distinct environmental cluster, and the admixture populations displayed heterogeneous environmental signatures. Collectively, these findings support local adaptation in *B. insignis*, driven by the interplay of environmental variation and geographic distance.

To evaluate the risk of genetic maladaptation under global warming, we employed a genetic offset framework^49,50^. Under various emission scenarios, higher greenhouse gas concentrations consistently produced larger offsets, signaling a greater risk of maladaptation (Supplementary Fig. 30). Populations in the southwestern Indochinese Peninsula exhibited relatively high local offsets, whereas populations outside this region showed lower offsets (Supplementary Fig. 31a, d). Forward and reverse offsets were generally very low across the species’ range, suggesting that future habitats may remain suitable (Supplementary Fig. 31b, c, e, f). However, these theoretical predictions must be interpreted in light of dispersal limitations, as *B. insignis* spores typically travel only tens to hundreds of kilometers^51^. Under high genetic offsets ( > 0.15), most populations are strongly locally adapted to future climates, with only a few in the Indochinese Peninsula requiring long-distance migration (Supplementary Fig. 32a). Considering moderate offsets ( > 0.05), the required migration distances increase by several thousand kilometers, with many populations needing to disperse over 5,000 km to avoid maladaptation, far exceeding their natural dispersal capacity (Supplementary Fig. 32b). Collectively, these results indicate that populations in the southwestern Indochinese Peninsula face elevated genetic vulnerability and high risk under future climate change (Fig. 5d). Polar plotting revealed no clear directional trend for potential migration (Supplementary Fig. 33), reinforcing the likelihood that range fragmentation, rather than large-scale migration, will shape the future distribution of *B. insignis*. Overall, these findings highlight not only the magnitude of genetic vulnerability but also the limited evolutionary and dispersal capacity of *B. insignis* under rapid climate change. The pronounced regional differences in offset levels suggest that conservation strategies may need to be spatially tailored, prioritizing populations in the southwestern Indochinese Peninsula. Moreover, the extreme migration distances implied by moderate offsets emphasize that natural dispersal alone is unlikely to maintain adaptive potential, raising the possibility that assisted gene flow or habitat connectivity restoration may be necessary to safeguard long-term persistence.

## Methods

### Plant materials and genome sequencing

Fresh fronds from a single *B. insignis* individual were collected from the South China National Botanical Garden in Guangzhou, China. Total genomic DNA was extracted using the CTAB method^52^. A preliminary genome survey was performed on the BGI DNBSEQ^TM^ platform, generating 512.37 Gb of short-read data to estimate genome size, heterozygosity, and repeat content. High-molecular-weight DNA libraries (15–18 kb) were constructed and sequenced on the PacBio Sequel II/IIe platform in CCS (HiFi) mode, generating 359.99 Gb of HiFi reads with an average read length of 16.54 kb. Hi-C libraries were prepared according to established protocols^53,54^. Briefly, fresh fronds were fixed with 4% formaldehyde, digested with DpnII, and biotin-labeled DNA ligation products were sheared and used to build Illumina PE150 libraries. Five Hi-C libraries were sequenced; each amplified for 12–14 PCR cycles.

### Genome size estimation and chromosome counting

A 17-mer frequency distribution was generated using KmerFreq (v4.0 in GCE v.1.0.2), and the genome size was calculated in GCE (v.1.0.2)^55^. Flow cytometry was performed for confirmation: leaf nuclei from *B. insignis* and the internal control *Camellia sinensis var. assamica*^56^ were co-stained with propidium iodide and analyzed using a BD FACScalibur flow cytometer at 488 nm excitation. The C-values were calculated based on the PI fluorescence peaks of the sample and control. For chromosome counting, root tips ( ~ 1 cm) from the sequenced individual were pretreated with p-dichlorobenzene for 5 h at room temperature, fixed in 3:1 (v/v) ethanol-to-acetic-acid solution at 4 °C, then macerated in 1 M HCl at 60 °C for 8 min. Chromosomes were stained with carbol fuchsin and observed by optical microscopy^57^.

### Genome assembly and quality assessment

Genome assembly was performed with Hifiasm (v.0.19.5-r587)^58^ in Hi-C mode using default parameters, incorporating both HiFi and Hi-C reads. The primary assembly was 8.78 Gb in length, consisting of 5,791 contigs (contig N50 of 4.36 Mb; Table 1). Hi-C reads were mapped to the primary assembly with BWA (v.0.7.17-r1188)^59^, scaffolding was performed using Juicer (v.1.6)^60^ and 3D-DNA pipeline^61^, followed by manual curation using Juicerbox module.

Assembly quality was evaluated by mapping short reads with BWA-MEM, completeness was assessed with BUSCO (viridiplantae_odb12 dataset)^62^. Additionally, N50 and other quality metrics were obtained using QUAST (v.5.2.0)^63^. Finally, CRAQ (v.1.0.9)^64^ was used to identify regional (R-AQI) and structural (S-AQI) assembly errors.

### Genome annotation

We employed an integrated approach of homology alignment and de novo search to annotate repetitive elements. RepeatMasker (http://www.repeatmasker.org) with Dfam^65^ and Repbase^66^ databases was employed for homology-based detection. For ab initio prediction, a de novo transposable element (TE) library was generated using MITE-Hunter^67^ and RepeatModeler (v.2.0.3)^68^. The unknown elements were classified by DeepTE^69^, and a non-redundant TE library was produced via Uclust^70^. Final repeats were masked with RepeatMasker.

Protein-coding genes were predicted using ab initio, homology-based, and RNA-seq–assisted approaches (details in Supplementary Note 1). Functional annotations were assigned via BLASTp (E-value ≤ 1e − 5) against Swiss-Prot^71^, with motifs and domains identified by InterProScan (v.5.39)^72^. Gene Ontology (GO) terms and Kyoto Encyclopedia of Genes and Genomes (KEGG) pathway annotations were assigned accordingly. Non-coding RNAs were characterized as described in the Supplementary Note 2.

### Comparative genomics analysis

Synteny and whole-genome duplication events were evaluated using WGDI (v0.6.1)^33^ for collinearity and *K*_s_ distributions. Substitution rate variation was accounted for by employing four species trios: (1) *B. insignis, Adiantum capillus-veneris, Lygodium japonicum*; (2) *B. insignis, Ceratopteris richardii, L. japonicum*; (3) *B. insignis, Alsophila spinulosa, L. japonicum*; and (4) *B. insignis, Marsilea vestita, L. japonicum*. We standardized the synonymous substitution rates for each trio from divergence events between species to the focal species (*B*. *insignis*)^73^.

For analyses of LTR retrotransposons (LTR-RTs), we obtained genomic data from seven available fern species—*Salvinia cucullata*^74^, *Azolla filiculoides*^74^, *Marsilea vestita*^75^, *Adiantum capillus-veneris*^29^, *Alsophila spinulosa*^28^, *Adiantum nelumbodies*^76^, and *Ceratopteris richardii*^27^—from publicly available datasets. LTR-RTs were identified uniformly in eight fern species using LTR_Finder^77^, LTRharvest^78^, and LTR_retriever^79^. We applied a phylogenetically informed calibration to account for lineage-specific rate heterogeneity^80^. For LTR retrotransposons, we estimated the corrected nucleotide substitutions (*K*) between paired 5’ and 3’ LTRs using LTR_retriever, which employs the Jukes-Cantor (JC69) model. The resulting *K* values were converted into LTR insertion times using lineage-specific substitution rates calibrated on a phylogenetic framework, enabling cross-species comparisons. The results were visualized in a density plot using ggplot2 (v.3.5.1)^81^.

To investigate evolutionary rates, we performed phylogenomic analyses on 16 representative fern species using 8,720 low-copy and 31 single-copy orthologs identified by Orthofinder (v.2.5.5)^82^. Multiple sequence alignments were performed in MAFFT (v.7.520)^83^, trimmed by trimAL (v.1.4.rev15)^84^. A maximum likelihood (ML) phylogeny was then inferred with IQ-TREE (v.2.2.6)^85^, employing 1,000 bootstrap replicates. Substitution rates (*d*_S_, *d*_N_) were estimated with PAML (v.4.8)^86^, and a likelihood ratio test compared free-ratio and one-ratio models. The significance of differences in synonymous (dS) and nonsynonymous (dN) substitution rates among the predefined groups (single-copy and low-copy) was evaluated using analysis of molecular variance (AMOVA). Finally, relative rate tests (RRT) were performed in MEGA^87^ on a concatenated protein alignment of 31 single-copy genes.

### Gene family identification and evolution

For gene family evolution, a phylogenetic tree was constructed across 16 taxa, including a bryophyte (*Physcomitrium patens*), a lycophyte (*Selaginella moellendorffii*), four seed plants, and ten ferns (including *B. insignis*). We rooted the resulting tree with *P. patens*, converted it into an ultrametric chronogram using r8s^88^, and calibrated it against TimeTree (v.5.0)^89^. CAFE (v.4.2.1)^90^ was used to identify significantly expanded or contracted gene families (*P* < 0.05). We characterized 11 gene families associated with the monolignol biosynthesis based on homology to *Alsophila spinulosa* genes. One-to-one orthologs in *B. insignis*, *A. spinulosa*, and *Sphaeropteris lepifera* were identified by reciprocal BLAST, and *K*_a_/*K*_s_ ratios were calculated in KaKs_Calculator (v.3.0)^91^ to assess selective pressures on lignin-related genes.

### Resequencing, SNP calling, and filtering

Fresh leaves from 94 *B. insignis* individuals representing 29 geographic locations (Supplementary Data 1) were used for DNA extraction using the CTAB method^52^. Paired-end libraries were sequenced on the DNBSEQ-T7 platform, and raw reads were quality-filtered with fastp (v.0.22.0)^92^. Clean reads were aligned to the *B. insignis* reference genome using BWA-MEM^59^. The resulting SAM files were converted to BAM format and sorted with SAMtools^93^. PCR duplicates were removed via Picard (https://broadinstitute.github.io/picard/). Variants were called following the GATK (v.4.5.0.0) pipeline^94^. Low-confidence variants were removed using the following strict hard-filtering thresholds: QD < 2.0 || MQ < 40.0 || FS > 60.0 || SOR > 3.0 || MQRankSum < -12.5 || ReadPosRankSum < -8.0. Subsequently, a multi-step soft-filtering process was applied to generate multiple high-quality SNP datasets for downstream analyses (Supplementary Fig. 15). We define the high-confidence variants (core variants) as biallelic SNPs retained after joint genotyping and stringent hard/soft filtering. All downstream variant panels (Dataset 1-3) were derived from this core variant set (Supplementary Fig. 15).

### Population structure and genetic diversity analyses

To assess population structure, we generated 342,582 unlinked SNPs (minor allele frequency > 5%), which were analyzed with ADMIXTURE (v.1.3.0)^95^ for K = 1 to K = 8 for cross-validation. Principal components analysis (PCA) was performed in PLINK (v.1.9)^96^, and a neighbor-joining (NJ) phylogenetic tree was constructed using PHYLIP. A haplotype network was inferred with PopART^97^ using the 95% statistical parsimony (TCS) network method (Supplementary Note 3). Nucleotide diversity (π), genetic differentiation coefficient (*F*_ST_) and Tajima’s *D* were estimated in 100-kb windows using pixy (v.1.2.10)^98^ and VCFTools (v.0.1.16)^99^. Linkage disequilibrium (LD) decay was calculated via PopLDdecay (v.3.42)^100^.

### Demographic history inference

For demographic history, we selected six samples ( ≥ 20× coverage) per major lineage to build diploid consensus sequences using BCFtools (v.1.14)^101^. PSMC^102^ was run with default parameters (25 iterations, -N25, -r5, -p 4 + 25*2 + 4 + 6) under a mutation rate of 1.25 × 10^−8^ per site per generation and a 5-year generation time. In addition, SMC + + (v.1.15.2)^103^ was performed to infer a finer-scale reconstruction of recent demographic histories within each lineage. To model complex demographic scenarios, we employed a stepwise, hierarchical approach using fastsimcoal2^104^. We initially modeled simpler histories with fewer parameters (e.g., divergence without migration), and progressively incorporated additional features such as continuous migration and pulse admixture. A total of 10 demographic and 5 differentiation models (Supplementary Fig. 21 and 22) were evaluated, each with 100 independent runs. The best-fitting scenario was selected based on the lowest AIC and ΔLhood (Supplementary Table 8–10). This stepwise strategy balances model realism with statistical robustness and mitigates potential identifiability issues arising from simultaneous estimation of many parameters.

To investigate gene flow, we generated a maximum likelihood drift tree using TreeMix (v.1.13)^105^ with migration edges from 1 to 5, selecting the optimal number with OptM^106^. Finally, identity-by-descent (IBD) segments were identified using the BEAGLE algorithm^107^.

### Estimation of inbreeding and genetic load

Individual heterozygosity was estimated with ANGSD (v. 0.937)^108^ under a folded site frequency spectrum (fSFS) model. Runs of homozygosity (ROHs) were identified with PLINK (v.1.9)^96^ using a minimum of 50 consecutive SNPs per 100-kb window. The inbreeding coefficient (*F*_ROH_) was calculated as the proportion of the genome covered by ROHs^109,110^.

Derived alleles were defined as those where more than 50% of individuals carried the same homozygous genotype^111^. SnpEff (v.4.3t)^112^ was used to annotate missense and loss-of-function (LOF) variants. Missense mutations were further assessed using SIFT-4G^113^ to classify potentially deleterious (DEL) substitutions, and the Grantham Score (GS ≥ 150)^114^ was applied to confirm deleterious missense variants. We calculated the proportion of homozygous DEL and LOF sites relative to the total number of homozygous plus heterozygous sites of each individual. Correlations among heterozygosity, inbreeding, and deleterious load (DEL and LOF) were visualized with SRplot^115^. We compared homozygous LOF variant frequencies inside and outside ROHs to evaluate the impact of inbreeding on genetic load. To quantify nonsynonymous and synonymous diversity, we identified 0- and 4-fold degenerate sites from the reference CDS using the degenotate pipline (https://github.com/harvardinformatics/degenotate), extracted the corresponding population polymorphism data from variant call format (VCF) file, and calculated nucleotide diversity (π) at these sites with ANGSD^108^.

### Selective sweep analysis

Selective sweeps were examined for two major lineages (YN and SC). We used a combined approach incorporating high *F*_ST_ signals (top 5% in 500 kb windows, 5 kb step) and within-lineage sweeps identified by RaiSD (v.2.9)^116^. Candidate selective sweeps were defined where both methods overlapped, with the 99.99% quantile of μ serving as the threshold. Manhattan plots were generated in R using the qqman package^117^.

### Genotype-environment association analyses

We retrieved 19 bioclimatic layers (1970–2000) from WorldClim (https://worldclim.org/) using ArcGIS v.10.8 for spatial extraction and retained seven uncorrelated variables (|*r*| < 0.7) via Pearson correlation: bio1, bio2, bio3, bio7, bio12, bio14, and bio15 (Supplementary Fig. 34). To identify a robust set of climate-adapted loci, we applied Latent Factor Mixed Models (LFMM) and Redundancy Analysis (RDA) to test genotype–environment associations (GEA). LFMM were conducted using three latent factors in the LEA package (v.3.16.0)^118^ to account for population structure. Variants significantly associated (FDR < 0.05) with at least three environmental variables were considered outliers. RDA was performed using vegan (v.2.6-8)^119^. Significant variants were identified based on extreme loadings (±3.5 SD, equivalent to a two-tailed *P* value of 0.0005) along one or more RDA axes.

To disentangle the effects of isolation by distance (IBD) from isolation by environment (IBE), we conducted Mantel and partial Mantel tests separately on adaptive and neutral SNP datasets. Pairwise matrices of *F*_ST_/(1 − *F*_ST_) were tested for correlation with geographic and environmental distances using the vegan package.

### Genetic offset under future climate scenarios

Genetic offset (also termed genomic vulnerability or risk of non-adaptedness, RONA^49,50^) was estimated specifically using the adaptive SNPs. We applied generalized dissimilarity modeling (GDM)^120,121^ with future climate projections (2081–2100) from WorldClim2.1 under two climate models (BCC-CSM2-MR and GISS-E2-1-G) and two Shared Socioeconomic Pathways (SSP245 and SSP585) at 2.5 arcminutes resolution. Because the two climate models showed a strong correlation (Supplementary Fig. 35), BCC-CSM2-MR was used. Forward and reverse genetic offsets^120,122^ were calculated to account for the potential effects of migration, then normalized across each channel (RGB) for spatial visualization. The local, forward, and reverse offsets were then mapped to the red, green, and blue channels, respectively. For each grid, we calculated genetic offsets and the maximum allowable migration distance and used Matplotlib (https://matplotlib.org/) to create a polar plot representing migration distance and direction.

### Reporting summary

Further information on research design is available in the Nature Portfolio Reporting Summary linked to this article.

## Supplementary information

Supplementary Informations

Description of Addiational Supplemenatry Files

Supplementary Data 1

Supplementary Data 2

Reporting Summary

Peer Review file

## Source data

Source Data

## Data Availability

Data supporting the findings of this work are available within the paper and its Supplementary Information files. The plant materials generated during the current study are available from the corresponding author upon request. All raw sequence datasets and genome assembly have been deposited at NCBI under the BioProject PRJNA1203580. The genome annotation files are available at Figshare (10.6084/m9.figshare.28229507). Source data are provided with this paper.

## References

[1] Kenrick, P. & Crane, P. R. The origin and early evolution of plants on land. *Nature* **389**, 33–39 (1997).

[2] Hassler, M. et al. Checklist of ferns and lycophytes of the world in the catalogue of life. Catalogue of Life, Amsterdam, Netherlands. 10.48580/dgjy9-3dc (2024).

[3] Kessler, M. et al. *Biogeography of Ferns* (Cambridge University Press, 2010).

[4] Page, C. N. Ecological strategies in fern evolution: a neopteridological overview. *Rev. Palaeobot. Palynol.* **119**, 1–33 (2002).

[5] Pryer, K. M. et al. Horsetails and ferns are a monophyletic group and the closest living relatives to seed plants. *Nature* **409**, 618–622 (2001). 11214320 10.1038/35054555

[6] Wolf, P. G., Schneider, H. & Ranker, T. Geographic distributions of homosporous ferns: does dispersal obscure evidence of vicariance? *J. Biogeogr.* **28**, 263–270 (2001).

[7] Niu, S. et al. The Chinese pine genome and methylome unveil key features of conifer evolution. *Cell* **185**, 204–217 (2022). 34965378 10.1016/j.cell.2021.12.006

[8] Neale, D. B. et al. Assembled and annotated 26.5 Gbp coast redwood genome: a resource for estimating evolutionary adaptive potential and investigating hexaploid origin. *G3 Genes|Genomes|Genet.* **12**, jkab380 (2022). 35100403 10.1093/g3journal/jkab380 PMC8728005

[9] Schartl, M. et al. The genomes of all lungfish inform on genome expansion and tetrapod evolution. *Nature* **634**, 96–103 (2024). 39143221 10.1038/s41586-024-07830-1 PMC11514621

[10] Wang, F. G. et al. Genome size evolution of the extant lycophytes and ferns. *Plant Divers* **44**, 141–152 (2022). 35505989 10.1016/j.pld.2021.11.007 PMC9043363

[11] Clark, J. et al. Genome evolution of ferns: evidence for relative stasis of genome size across the fern phylogeny. *N. Phytol.* **210**, 1072–1082 (2016). 10.1111/nph.13833 26756823

[12] Fernández, P. et al. A 160 Gbp fork fern genome shatters size record for eukaryotes. *iScience* **27**, 109889 (2024). 39055604 10.1016/j.isci.2024.109889 PMC11270024

[13] Bourke, P. M., Voorrips, R. E., Visser, R. G. & Maliepaard, C. Tools for genetic studies in experimental populations of polyploids. *Front. Plant Sci.* **9**, 513 (2018). 29720992 10.3389/fpls.2018.00513 PMC5915555

[14] Dufresne, F., Stift, M., Vergilino, R. & Mable, B. K. Recent progress and challenges in population genetics of polyploid organisms: an overview of current state-of-the-art molecular and statistical tools. *Mol. Ecol.* **23**, 40–69 (2014). 24188632 10.1111/mec.12581

[15] Klekowski, E. J. Jr & Baker, H. G. Evolutionary significance of polyploidy in the Pteridophyta. *Science* **153**, 305–307 (1966). 17780002 10.1126/science.153.3733.305

[16] PPGI A community-derived classification for extant lycophytes and ferns. *J. Syst. Evol.* **54**, 563–603 (2016).

[17] Sun, L. et al. Forest diversity and vitality of the important relict and endangered fern species, *Brainea insignis* in China. *J. Trop. Sci.* **33**, 356–367 (2021).

[18] Wu, P., Xie, H., Tao, W., Miao, S. & Wei, X. Phytoecdysteroids from the rhizomes of *Brainea insignis*. *Phytochemistry* **71**, 975–981 (2010). 20347104 10.1016/j.phytochem.2010.03.002

[19] Fang, Y. S. et al. Chemical constituents from the fern *Brainea insignis* (Blechnaceae). *Plant Div.* **30**, 725 (2008).

[20] Liu, H. et al. Development and characterization of EST-SSR markers via transcriptome sequencing in *Brainea insignis* (Aspleniaceae s.l.). *Appl. Plant Sci.* **5**, 1700067 (2017). 10.3732/apps.1700067 PMC5664966 29109921

[21] Kholia, B., Sharma, S. & Sinha, B. *Brainea insignis* (Hook.) J. Sm.–a conservation priority fern of North East India. *Curr. Sci.* **116**, 32–34 (2019).

[22] Raffaelli, D. How extinction patterns affect ecosystems. *Science* **306**, 1141–1142 (2004). 15539589 10.1126/science.1106365

[23] Wolf, P. G. et al. An exploration into fern genome space. *Mol. Biol. Evol.* **7**, 2533–2544 (2015). 10.1093/gbe/evv163 PMC4607520 26311176

[24] Baniaga, A. E. & Barker, M. S. Nuclear genome size is positively correlated with median LTR-RT insertion time in fern and lycophyte genomes. *Am. Fern J.* **109**, 248–266 (2019).

[25] Zhang, G. et al. The reference genome of *Miscanthus floridulus* illuminates the evolution of Saccharinae. *Nat. Plants* **7**, 608–618 (2021). 33958777 10.1038/s41477-021-00908-y PMC8238680

[26] Schmutz, J. et al. Genome sequence of the palaeopolyploid soybean. *Nature* **463**, 178–183 (2010). 20075913 10.1038/nature08670

[27] Marchant, D. B. et al. Dynamic genome evolution in a model fern. *Nat. Plants* **8**, 1038–1051 (2022). 36050461 10.1038/s41477-022-01226-7 PMC9477723

[28] Huang, X. et al. The flying spider-monkey tree fern genome provides insights into fern evolution and arborescence. *Nat. Plants* **8**, 500–512 (2022). 35534720 10.1038/s41477-022-01146-6 PMC9122828

[29] Fang, Y. et al. The genome of homosporous maidenhair fern sheds light on the euphyllophyte evolution and defences. *Nat. Plants* **8**, 1024–1037 (2022). 36050462 10.1038/s41477-022-01222-x PMC7613604

[30] Wickell, D. et al. Underwater CAM photosynthesis elucidated by *Isoetes* genome. *Nat. Commun.* **12**, 6348 (2021). 34732722 10.1038/s41467-021-26644-7 PMC8566536

[31] Li, C. et al. Extraordinary preservation of gene collinearity over three hundred million years revealed in homosporous lycophytes. *Proc. Natl. Acad. Sci. USA* **121**, e2312607121 (2024). 38236735 10.1073/pnas.2312607121 PMC10823260

[32] Sensalari, C., Maere, S. & Lohaus, R. ksrates: positioning whole-genome duplications relative to speciation events in *K*_s_ distributions. *Bioinformatics* **38**, 530–532 (2022). 34406368 10.1093/bioinformatics/btab602

[33] Sun, P. et al. WGDI: a user-friendly toolkit for evolutionary analyses of whole-genome duplications and ancestral karyotypes. *Mol. Plant* **15**, 1841–1851 (2022). 36307977 10.1016/j.molp.2022.10.018

[34] Wendel, J. F., Jackson, S. A., Meyers, B. C. & Wing, R. A. Evolution of plant genome architecture. *Genome Biol.* **17**, 37 (2016). 26926526 10.1186/s13059-016-0908-1 PMC4772531

[35] Michael, T. P. Plant genome size variation: bloating and purging DNA. *Brief. Funct. Genomics* **13**, 308–317 (2014). 24651721 10.1093/bfgp/elu005

[36] Ali, Z. M. et al. Comparative transcriptomics in ferns reveals key innovations and divergent evolution of the secondary cell walls. *Nat. Plants* **11**, 1028–1048 (2025). 40269175 10.1038/s41477-025-01978-y

[37] Lisiecki, L. E. & Raymo, M. E. A Pliocene-Pleistocene stack of 57 globally distributed benthic δ18O records. *Paleoceanography* **20**, PA1003 (2005).

[38] EPICA-community-members Eight glacial cycles from an Antarctic ice core. *Nature* **429**, 623–628 (2004). 15190344 10.1038/nature02599

[39] Zhao, Y. P. et al. Resequencing 545 ginkgo genomes across the world reveals the evolutionary history of the living fossil. *Nat. Commun.* **10**, 4201 (2019). 31519986 10.1038/s41467-019-12133-5 PMC6744486

[40] Zhu, S. et al. Genomic insights on the contribution of balancing selection and local adaptation to the long-term survival of a widespread living fossil tree, *Cercidiphyllum japonicum*. *N. Phytol.* **228**, 1674–1689 (2020). 10.1111/nph.16798 32643803

[41] Chen, Y. et al. Genomic analyses of a “living fossil”: The endangered dove-tree. *Mol. Ecol. Resour.* **20**, 756–769 (2020). 10.1111/1755-0998.13138 31970919

[42] Yi, H., Wang, J., Dong, S. & Kang, M. Genomic signatures of inbreeding and mutation load in tree ferns. *Plant J.* **120**, 1522–1535 (2024). 39387366 10.1111/tpj.17064

[43] Yang, Y. et al. Genomic effects of population collapse in a critically endangered ironwood tree *Ostrya rehderiana*. *Nat. Commun.* **9**, 5449 (2018). 30575743 10.1038/s41467-018-07913-4 PMC6303402

[44] Xue, Y. et al. *Mountain gorilla* genomes reveal the impact of long-term population decline and inbreeding. *Science* **348**, 242–245 (2015). 25859046 10.1126/science.aaa3952 PMC4668944

[45] Xie, H. X. et al. Ancient demographics determine the effectiveness of genetic purging in endangered lizards. *Mol. Biol. Evol.* **39**, msab359 (2022). 34919713 10.1093/molbev/msab359 PMC8788223

[46] Dussex, N. et al. Population genomics of the critically endangered kākāpō. *Cell Genom.* **1**, 100002 (2021). 36777713 10.1016/j.xgen.2021.100002 PMC9903828

[47] Grossen, C., Guillaume, F., Keller, L. F. & Croll, D. Purging of highly deleterious mutations through severe bottlenecks in *Alpine ibex*. *Nat. Commun.* **11**, 1001 (2020). 32081890 10.1038/s41467-020-14803-1 PMC7035315

[48] Robinson, J. A., Brown, C., Kim, B. Y., Lohmueller, K. E. & Wayne, R. K. Purging of strongly deleterious mutations explains long-term persistence and absence of inbreeding depression in island foxes. *Curr. Biol.* **28**, 3487–3494. e4 (2018). 30415705 10.1016/j.cub.2018.08.066 PMC6462144

[49] Rellstab, C. Genomics helps to predict maladaptation to climate change. *Nat. Clim. Chang.* **11**, 85–86 (2021).

[50] Fitzpatrick, M. C. & Keller, S. R. Ecological genomics meets community-level modelling of biodiversity: Mapping the genomic landscape of current and future environmental adaptation. *Ecol. Lett.* **18**, 1–16 (2015). 25270536 10.1111/ele.12376

[51] Sheffield, E. From pteridophyte spore to sporophyte in the natural environment. In *Pteridology in Perspective* (eds. Camus, J. M., Gibby, M. & Johns, R. J.) 541–549 (Royal Botanic Gardens, Kew, 1996).

[52] Porebski, S., Bailey, L. G. & Baum, B. R. Modification of a CTAB DNA extraction protocol for plants containing high polysaccharide and polyphenol components. *Plant Mol. Biol. Rep.* **15**, 8–15 (1997).

[53] Rao, S. S. et al. A 3D map of the human genome at kilobase resolution reveals principles of chromatin looping. *Cell* **159**, 1665–1680 (2014). 25497547 10.1016/j.cell.2014.11.021 PMC5635824

[54] Van Berkum, N. L. et al. Hi-C: a method to study the three-dimensional architecture of genomes. *J*. *Vis*. *Exp*. **6**, e1869 (2010). 10.3791/1869 PMC3149993 20461051

[55] Liu, B. et al. Estimation of genomic characteristics by analyzing k-mer frequency in de novo genome projects. *Quant. Biol.* **35**, 62–67 (2013).

[56] Wei, C. et al. Draft genome sequence of *Camellia sinensis var. sinensis* provides insights into the evolution of the tea genome and tea quality. *Proc. Natl. Acad. Sci. USA* **115**, E4151–E4158 (2018). 29678829 10.1073/pnas.1719622115 PMC5939082

[57] Zhang, R. et al. Dating whole genome duplication in *Ceratopteris thalictroides* and potential adaptive values of retained gene duplicates. *Int. J. Mol. Sci.* **20**, 1926 (2019). 31010109 10.3390/ijms20081926 PMC6515051

[58] Cheng, H. et al. Haplotype-resolved assembly of diploid genomes without parental data. *Nat. Biotechnol.* **40**, 1332–1335 (2022). 35332338 10.1038/s41587-022-01261-x PMC9464699

[59] Li, H. & Durbin, R. Fast and accurate short read alignment with Burrows–Wheeler transform. *Bioinformatics* **25**, 1754–1760 (2009). 19451168 10.1093/bioinformatics/btp324 PMC2705234

[60] Durand, N. C. et al. Juicer provides a one-click system for analyzing loop-resolution Hi-C experiments. *Cell Syst.* **3**, 95–98 (2016). 27467249 10.1016/j.cels.2016.07.002 PMC5846465

[61] Dudchenko, O. et al. De novo assembly of the *Aedes aegypti* genome using Hi-C yields chromosome-length scaffolds. *Science* **356**, 92–95 (2017). 28336562 10.1126/science.aal3327 PMC5635820

[62] Simão, F. A., Waterhouse, R. M., Ioannidis, P., Kriventseva, E. V. & Zdobnov, E. M. BUSCO: assessing genome assembly and annotation completeness with single-copy orthologs. *Bioinformatics* **31**, 3210–3212 (2015). 26059717 10.1093/bioinformatics/btv351

[63] Gurevich, A., Saveliev, V., Vyahhi, N. & Tesler, G. QUAST: quality assessment tool for genome assemblies. *Bioinformatics* **29**, 1072–1075 (2013). 23422339 10.1093/bioinformatics/btt086 PMC3624806

[64] Li, K., Xu, P., Wang, J., Yi, X. & Jiao, Y. Identification of errors in draft genome assemblies at single-nucleotide resolution for quality assessment and improvement. *Nat. Commun.* **14**, 6556 (2023). 37848433 10.1038/s41467-023-42336-w PMC10582259

[65] Wheeler, T. J. et al. Dfam: a database of repetitive DNA based on profile hidden Markov models. *Nucleic Acids Res*. **41**, D70–D82 (2012). 23203985 10.1093/nar/gks1265 PMC3531169

[66] Jurka, J. et al. Repbase Update, a database of eukaryotic repetitive elements. *Cytogenet. Genome Res.* **110**, 462–467 (2005). 16093699 10.1159/000084979

[67] Han, Y. & Wessler, S. R. MITE-Hunter: a program for discovering miniature inverted-repeat transposable elements from genomic sequences. *Nucleic Acids Res* **38**, e199–e199 (2010). 20880995 10.1093/nar/gkq862 PMC3001096

[68] Flynn, J. M. et al. RepeatModeler2 for automated genomic discovery of transposable element families. *Proc. Natl. Acad. Sci. USA* **117**, 9451–9457 (2020). 32300014 10.1073/pnas.1921046117 PMC7196820

[69] Yan, H., Bombarely, A. & Li, S. DeepTE: a computational method for de novo classification of transposons with convolutional neural network. *Bioinformatics* **36**, 4269–4275 (2020). 32415954 10.1093/bioinformatics/btaa519

[70] Edgar, R. C. Search and clustering orders of magnitude faster than BLAST. *Bioinformatics* **26**, 2460–2461 (2010). 20709691 10.1093/bioinformatics/btq461

[71] Bairoch, A. & Apweiler, R. The SWISS-PROT protein sequence database and its supplement TrEMBL in 2000. *Nucleic Acids Res* **28**, 45–48 (2000). 10592178 10.1093/nar/28.1.45 PMC102476

[72] Jones, P. et al. InterProScan 5: genome-scale protein function classification. *Bioinformatics* **30**, 1236–1240 (2014). 24451626 10.1093/bioinformatics/btu031 PMC3998142

[73] Chen, H. et al. Revisiting ancient polyploidy in leptosporangiate ferns. *N. Phytol.* **237**, 1405–1417 (2023). 10.1111/nph.18607 PMC7614084 36349406

[74] Li, F. W. et al. Fern genomes elucidate land plant evolution and cyanobacterial symbioses. *Nat. Plants* **4**, 460–472 (2018). 29967517 10.1038/s41477-018-0188-8 PMC6786969

[75] Rahmatpour, N. et al. Analyses of *Marsilea vestita* genome and transcriptomes do not support widespread intron retention during spermatogenesis. *N. Phytol.* **237**, 1490–1494 (2023). 10.1111/nph.18652 36451524

[76] Zhong, Y. et al. Genomic insights into genetic diploidization in the homosporous fern *Adiantum nelumboides*. *Genome Biol. Evol.* **14**, evac127 (2022). 35946426 10.1093/gbe/evac127 PMC9387920

[77] Xu, Z. & Wang, H. LTR_FINDER: an efficient tool for the prediction of full-length LTR retrotransposons. *Nucleic Acids Res*. **35**, W265–W268 (2007). 17485477 10.1093/nar/gkm286 PMC1933203

[78] Ellinghaus, D., Kurtz, S. & Willhoeft, U. LTRharvest, an efficient and flexible software for de novo detection of LTR retrotransposons. *BMC Bioinforma.* **9**, 18 (2008). 10.1186/1471-2105-9-18 PMC2253517 18194517

[79] Ou, S. & Jiang, N. LTR_retriever: a highly accurate and sensitive program for identification of long terminal repeat retrotransposons. *Plant Physiol.* **176**, 1410–1422 (2018). 29233850 10.1104/pp.17.01310 PMC5813529

[80] Chen, H. C., Zwaenepoel, A. & Van de Peer, Y. wgd v2: a suite of tools to uncover and date ancient polyploidy and whole-genome duplication. *Bioinformatics* **40**, btae272 (2024). 38632086 10.1093/bioinformatics/btae272 PMC11078771

[81] Wickham, H., Chang, W. & Wickham, M. H. *Package “ggplot2”. Create Elegant Data Visualisations Using the Grammar of Graphics*. **2**, 1–189 (2016).

[82] Emms, D. M. & Kelly, S. OrthoFinder: phylogenetic orthology inference for comparative genomics. *Genome Biol.* **20**, 238 (2019). 31727128 10.1186/s13059-019-1832-y PMC6857279

[83] Katoh, K., Misawa, K., Kuma, K. I. & Miyata, T. MAFFT: a novel method for rapid multiple sequence alignment based on fast Fourier transform. *Nucleic Acids Res* **30**, 3059–3066 (2002). 12136088 10.1093/nar/gkf436 PMC135756

[84] Capella-Gutiérrez, S., Silla-Martínez, J. M. & Gabaldón, T. trimAl: a tool for automated alignment trimming in large-scale phylogenetic analyses. *Bioinformatics* **25**, 1972–1973 (2009). 19505945 10.1093/bioinformatics/btp348 PMC2712344

[85] Nguyen, L.-T., Schmidt, H. A., Von Haeseler, A. & Minh, B. Q. IQ-TREE: a fast and effective stochastic algorithm for estimating maximum-likelihood phylogenies. *Mol. Biol. Evol.* **32**, 268–274 (2015). 25371430 10.1093/molbev/msu300 PMC4271533

[86] Yang, Z. PAML 4: phylogenetic analysis by maximum likelihood. *Mol. Biol. Evol.* **24**, 1586–1591 (2007). 17483113 10.1093/molbev/msm088

[87] Kumar, S., Stecher, G. & Tamura, K. MEGA7: molecular evolutionary genetics analysis version 7.0 for bigger datasets. *Mol. Biol. Evol.* **33**, 1870–1874 (2016). 27004904 10.1093/molbev/msw054 PMC8210823

[88] Sanderson, M. J. r8s: inferring absolute rates of molecular evolution and divergence times in the absence of a molecular clock. *Bioinformatics* **19**, 301–302 (2003). 12538260 10.1093/bioinformatics/19.2.301

[89] Kumar, S. et al. TimeTree 5: an expanded resource for species divergence times. *Mol. Biol. Evol.* **39**, msac174 (2022). 35932227 10.1093/molbev/msac174 PMC9400175

[90] De Bie, T., Cristianini, N., Demuth, J. P. & Hahn, M. W. CAFE: a computational tool for the study of gene family evolution. *Bioinformatics* **22**, 1269–1271 (2006). 16543274 10.1093/bioinformatics/btl097

[91] Zhang, Z. KaKs_Calculator 3.0: calculating selective pressure on coding and non-coding sequences. *Genom. Proteom. Bioinform.* **20**, 536–540 (2022). 10.1016/j.gpb.2021.12.002 PMC9801026 34990803

[92] Chen, S., Zhou, Y., Chen, Y. & Gu, J. Fastp: an ultra-fast all-in-one FASTQ preprocessor. *Bioinformatics* **34**, i884–i890 (2018). 30423086 10.1093/bioinformatics/bty560 PMC6129281

[93] Li, H. et al. The sequence alignment/map format and SAMtools. *Bioinformatics* **25**, 2078–2079 (2009). 19505943 10.1093/bioinformatics/btp352 PMC2723002

[94] McKenna, A. et al. The Genome Analysis Toolkit: a MapReduce framework for analyzing next-generation DNA sequencing data. *Genome Res* **20**, 1297–1303 (2010). 20644199 10.1101/gr.107524.110 PMC2928508

[95] Alexander, D. H., Novembre, J. & Lange, K. Fast model-based estimation of ancestry in unrelated individuals. *Genome Res* **19**, 1655–1664 (2009). 19648217 10.1101/gr.094052.109 PMC2752134

[96] Purcell, S. et al. PLINK: a tool set for whole-genome association and population-based linkage analyses. *Am. J. Hum. Genet.* **81**, 559–575 (2007). 17701901 10.1086/519795 PMC1950838

[97] Leigh, J. W., Bryant, D. & Nakagawa, S. POPART: full-feature software for haplotype network construction. *Methods Ecol. Evol.* **6**, 1110–1116 (2015).

[98] Korunes, K. L. & Samuk, K. pixy: Unbiased estimation of nucleotide diversity and divergence in the presence of missing data. *Mol. Ecol. Resour.* **21**, 1359–1368 (2021). 33453139 10.1111/1755-0998.13326 PMC8044049

[99] Danecek, P. et al. The variant call format and VCFtools. *Bioinformatics* **27**, 2156–2158 (2011). 21653522 10.1093/bioinformatics/btr330 PMC3137218

[100] Zhang, C., Dong, S. S., Xu, J. Y., He, W. M. & Yang, T. L. PopLDdecay: a fast and effective tool for linkage disequilibrium decay analysis based on variant call format files. *Bioinformatics* **35**, 1786–1788 (2019). 30321304 10.1093/bioinformatics/bty875

[101] Danecek, P. et al. Twelve years of SAMtools and BCFtools. *Gigascience* **10**, giab008 (2021). 33590861 10.1093/gigascience/giab008 PMC7931819

[102] Li, H. & Durbin, R. Inference of human population history from individual whole-genome sequences. *Nature* **475**, 493–496 (2011). 21753753 10.1038/nature10231 PMC3154645

[103] Schiffels, S. & Durbin, R. Inferring human population size and separation history from multiple genome sequences. *Nat. Genet.* **46**, 919–925 (2014). 24952747 10.1038/ng.3015 PMC4116295

[104] Excoffier, L. et al. fastsimcoal2: demographic inference under complex evolutionary scenarios. *Bioinformatics* **37**, 4882–4885 (2021). 34164653 10.1093/bioinformatics/btab468 PMC8665742

[105] Pickrell, J. & Pritchard, J. Inference of population splits and mixtures from genome-wide allele frequency data. *PLOS Genet* **8**, e1002967 (2012). 23166502 10.1371/journal.pgen.1002967 PMC3499260

[106] Fitak, R. R. OptM: estimating the optimal number of migration edges on population trees using Treemix. *Biol. Methods Protoc.* **6**, bpab017 (2021). 34595352 10.1093/biomethods/bpab017 PMC8476930

[107] Browning, B. L. & Browning, S. R. Improving the accuracy and efficiency of identity-by-descent detection in population data. *Genetics* **194**, 459–471 (2013). 23535385 10.1534/genetics.113.150029 PMC3664855

[108] Korneliussen, T. S., Albrechtsen, A. & Nielsen, R. ANGSD: analysis of next generation sequencing data. *BMC Bioinforma.* **15**, 356 (2014). 10.1186/s12859-014-0356-4 PMC4248462 25420514

[109] Curik, I., Ferenčaković, M. & Sölkner, J. Inbreeding and runs of homozygosity: a possible solution to an old problem. *Livest. Sci.* **166**, 26–34 (2014).

[110] McQuillan, R. et al. Runs of homozygosity in European populations. *Am. J. Hum. Genet.* **83**, 359–372 (2008). 18760389 10.1016/j.ajhg.2008.08.007 PMC2556426

[111] Hacia, J. G. et al. Determination of ancestral alleles for human single-nucleotide polymorphisms using high-density oligonucleotide arrays. *Nat. Genet.* **22**, 164–167 (1999). 10369258 10.1038/9674

[112] Cingolani, P. et al. A program for annotating and predicting the effects of single nucleotide polymorphisms, SnpEff. *Fly* **6**, 80–92 (2012). 22728672 10.4161/fly.19695 PMC3679285

[113] Vaser, R., Adusumalli, S., Leng, S. N., Sikic, M. & Ng, P. C. SIFT missense predictions for genomes. *Nat. Protoc.* **11**, 1–9 (2016). 26633127 10.1038/nprot.2015.123

[114] Grantham, R. Amino acid difference formula to help explain protein evolution. *Science* **185**, 862–864 (1974). 4843792 10.1126/science.185.4154.862

[115] Tang, D. et al. SRplot: A free online platform for data visualization and graphing. *PLoS One* **18**, e0294236 (2023). 37943830 10.1371/journal.pone.0294236 PMC10635526

[116] Alachiotis, N. & Pavlidis, P. RAiSD detects positive selection based on multiple signatures of a selective sweep and SNP vectors. *Commun. Biol.* **1**, 79 (2018). 30271960 10.1038/s42003-018-0085-8 PMC6123745

[117] Turner, S. D. qqman: an R package for visualizing GWAS results using Q-Q and manhattan plots. *J. Open Source Softw.* **3**, 1731 (2018).

[118] Frichot, E. & François, O. L. E. A. An R package for landscape and ecological association studies. *Methods Ecol. Evol.* **6**, 925–929 (2015).

[119] Dixon, P. VEGAN, a package of R functions for community ecology. *J. Veg. Sci.* **14**, 927–930 (2003).

[120] Gougherty, A. V., Keller, S. R. & Fitzpatrick, M. C. Maladaptation, migration and extirpation fuel climate change risk in a forest tree species. *Nat. Clim. Chang.* **11**, 166–171 (2021).

[121] Ferrier, S., Manion, G., Elith, J. & Richardson, K. Using generalized dissimilarity modelling to analyse and predict patterns of beta diversity in regional biodiversity assessment. *Divers. Distrib.* **13**, 252–264 (2007).

[122] Sang, Y. et al. Genomic insights into local adaptation and future climate-induced vulnerability of a keystone forest tree in East Asia. *Nat. Commun.* **13**, 6541 (2022). 36319648 10.1038/s41467-022-34206-8 PMC9626627

