# Supplementary material for: Decoding the genome of Brainea insignis reveals insights into fern evolution and conservation
Source: Nat Commun. 2025 Dec 30;17:1292. doi: 10.1038/s41467-025-68053-0 (PMC12868710; doi:10.1038/s41467-025-68053-0)
Supplement: Supplementary file 1 — Supplementary Informations [file 41467_2025_68053_MOESM1_ESM.pdf]

Decoding the genome of *Brainea insignis* reveals insights into fern evolution and conservation

Xia *et al.*

Supplementary Note 1. Genome structure annotation

The structural annotation of the genome was carried out using three complementary methods: ab initio prediction, homology-based prediction, and RNA-Seq-assisted prediction, aiming to accurately identify protein-coding gene models. Homology-based Prediction: Protein sequences from five species—*Adiantum capillus-veneris*[1], *Ceratopteris richardii*[2], *Alsophila spinulosa*[3], *Marsilea vestita*[4], and *Sphaeropteris lepifera*[5]—were used for homology-based annotation. The sequences were aligned to the target genome using TBLASTN (v.2.5.0+)[6] with an E-value threshold of ≤ 1e−5. GeneWise (v.2.4.1)[7] was then used for precise gene structure alignment, ensuring accurate homology-based gene prediction. Ab Initio Prediction: *De novo* gene prediction tools, AUGUSTUS[8] and SNAP[9] were utilized in the automated gene prediction pipeline to predict gene models, providing initial gene structure insights independent of external evidence. RNA-Seq Assisted Prediction: Six transcriptomes were generated from various tissues and growth phases, including gametophytes, roots, stems, curled leaves, developing leaves, and mature leaves. RNA-Seq reads from these tissues were aligned to the genome using HISAT[10] to identify exon regions and splice junctions. StringTie[11] was subsequently used for genome-based transcript assembly. Finally, the non-redundant reference gene set was generated by merging genes predicted by three methods with EvidenceModeler (EVM) (v.1.1.1)[12] incorporating Program to Assemble Spliced Alignment (PASA) terminal exon support and including masked transposable elements as input into gene prediction.

Supplementary Note 2. Non-coding RNA annotation

The annotation of non-coding RNAs (ncRNAs), including rRNAs, tRNAs, snRNAs, and miRNAs, was performed using specialized tools. Ribosomal RNAs (rRNAs): Identified using BLAST+ (v.2.5.0)[6] with an E-value threshold of ≤1e−10 against the Rfam (v.14.1)[13] database. Parameters were set to -v 10000 -b 10000 to enhance sensitivity. Small nuclear RNAs (snRNAs) and microRNAs (miRNAs): Detected using Infernal's cmscan tool (v.1.1.4)[14] by comparing sequence and secondary structure profiles against the Rfam database. Transfer RNAs (tRNAs): Predicted using tRNAscan-SE (v.1.4)[15], which identifies tRNA genes based on sequence and structural characteristics.

Supplementary Note 3. Chloroplast genome analysis

We assembled the chloroplast genome of *Brainea insignis* using GetOrganelle (v.1.7.7.0)[16] software with default settings. For improved annotation reliability, we used PGA[17] and the Geneious[18] platform for annotation analyses. Reads from 94 individuals were mapped to the assembled chloroplast genome using BWA (v. 0.7.17-r1188)[19] with the BWA-MEM algorithm. Variant calling was performed using BCFtools (v.1.14)[20], combining the "mpileup" and "call" commands to obtain SNPs in Variant Call Format (VCF). To distinguish between plastid and nuclear sequences, we used VCFtools (v.0.1.16)[21] to set a higher coverage threshold, specifically greater than 200 times (--min-meanDP 2000, --minDP 2000) the average sequencing depth of the genome, ensuring that chloroplast variants were retained with maximum likelihood. Positions not meeting these criteria were treated as missing data, and InDels were excluded from further analyses. The consensus sequence for each species was extracted from the VCF file using the "consensus" module in BCFtools, followed by alignment with MAFFT (v.7.520)[22]. Haplotypes for all individuals were then identified using DnaSP (v.6)[23], and a haplotype network was generated using PopArt (v.1.7)[24].

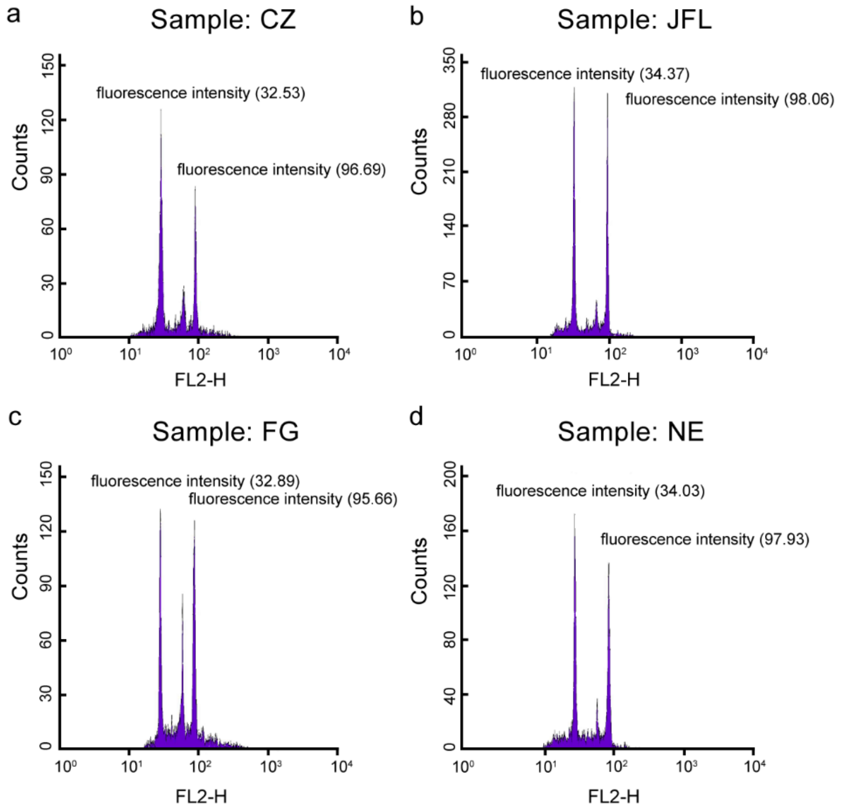

Supplementary Fig. 1. Flow cytometry analysis. Flow cytometry fluorescence analysis of four samples, where the first peak represents the internal control and the second peak corresponds to the target sample. Fluorescence intensity was used to assess the relative fluorescence signal of the target sample. **a,** Sample from Chongzuo, Guangxi, China (CZ). **b,** Sample from 'Jianfengling' mountain, Hainan, China (JFL). **c,** Sample from the Fern Garden of South China Botanical Garden (FG). **d,** Sample from Ninger, Yunnan, China (NE).

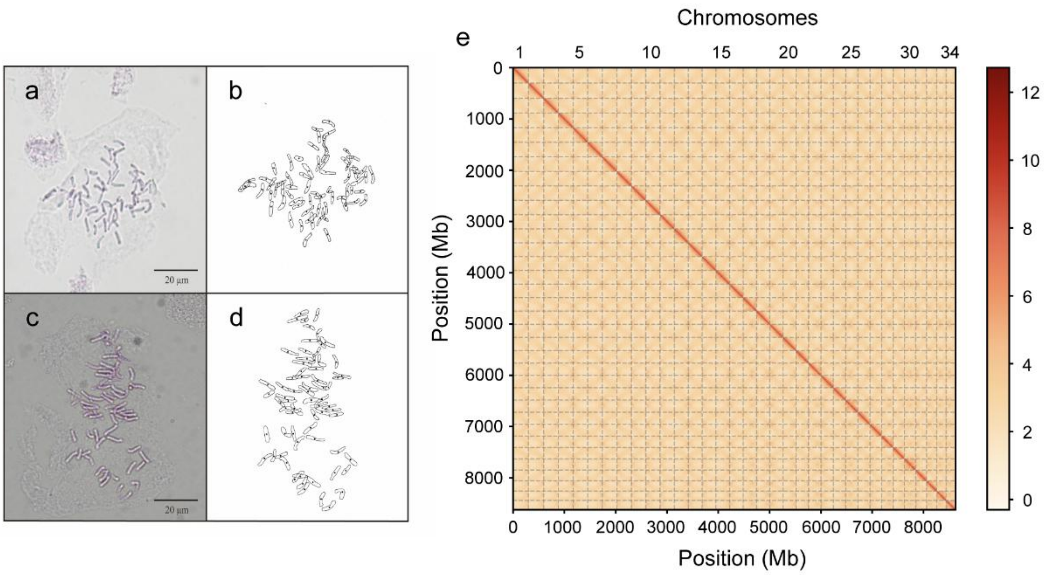

Supplementary Fig. 2. Chromosomes of B. insignis in mitotic root-tip cells (scale bars = 20 μm) and Hi-C map. a, Metaphase chromosomes of cell 1, diploid, 2n = 68. **b,** A schematic drawing of the chromosomes shown in panel a. **c,** Metaphase chromosomes of cell 2, diploid, 2n = 68. **d,** A schematic drawing of the chromosomes shown in panel c. **e,** Hi-C map showing genome-wide all-by-all interactions between 34 chromosomes.

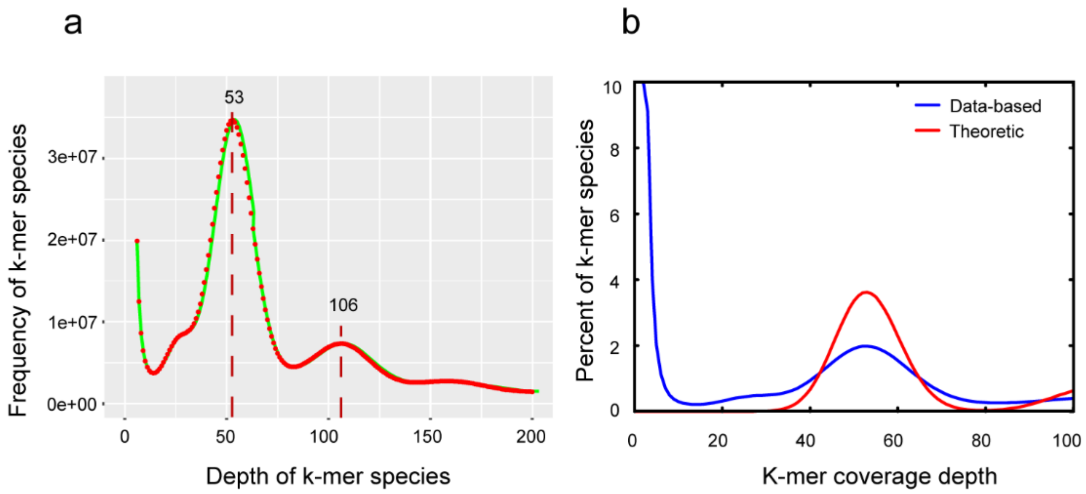

Supplementary Fig. 3. Genome survey of *B. insignis*. a, Genome survey distribution plot for *k*-mer size 17. The plot features a major peak at depth 53, representing the core genome sequence, and a secondary peak at depth 106, corresponding to repetitive sequences. **b**, *K*-mer distribution plot showing both data-based and theoretical distributions. The major peaks for both distributions align at the same position, indicating consistency between the observed and expected k-mer frequencies and supporting the reliability of survey.

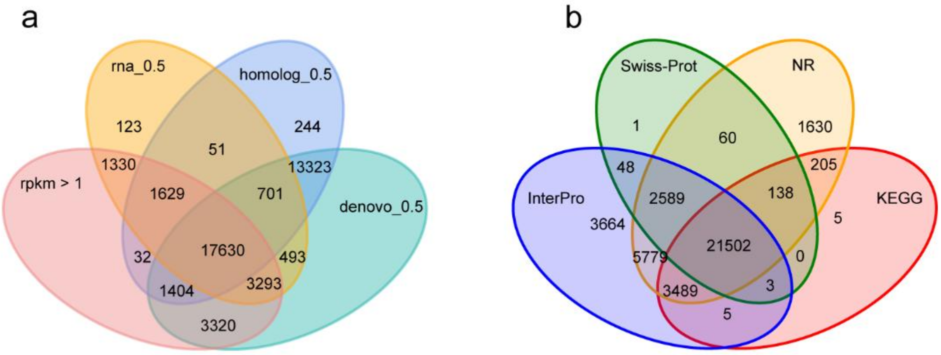

Supplementary Fig. 4. Genome annotation of *B. insignis*. a, Venn diagram showing the number of genes annotated using different methods. 'denovo' represents genes supported by *de novo* prediction integrated with EVM; 'homolog' represents genes supported by homologous prediction integrated with EVM; 'rna' represents genes supported by RNA-seq data integrated with EVM. Each evidence type is considered supported if gene overlap is greater than 50%. The numbers indicate the total count of genes in each category. **b**, The Venn diagram illustrates the overlap and unique gene functions assigned by different database, highlighting the consistency and divergence in functional annotation between them.

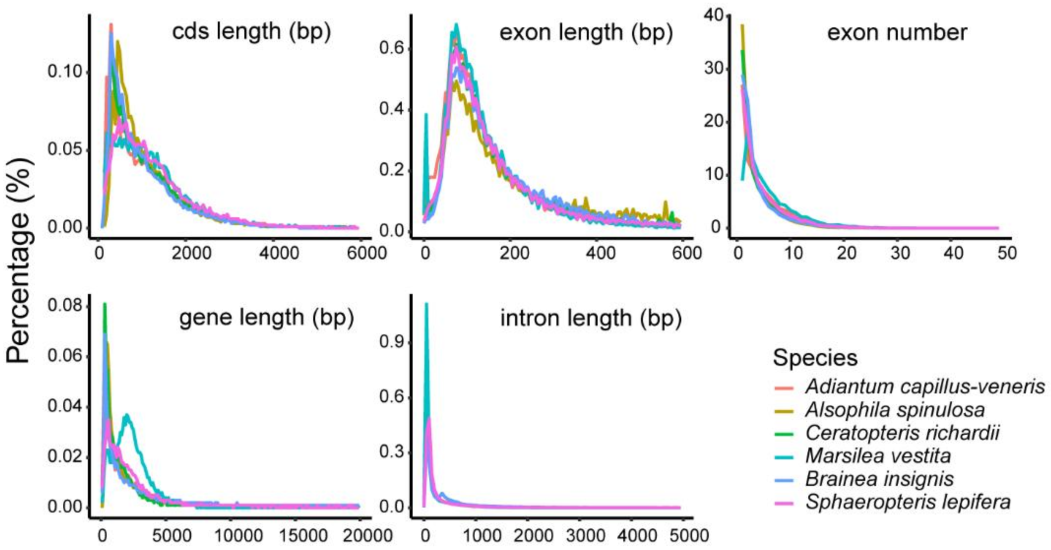

Supplementary Fig. 5. Comparison of genome structure characteristics. Comparison of gene structural features between *B*. *insignis* and other fern species, including CDS length, exon length, exon number, gene length, and intron length. The plot highlights similarities in the genomic architecture of *B*. *insignis* relative to other fern species.

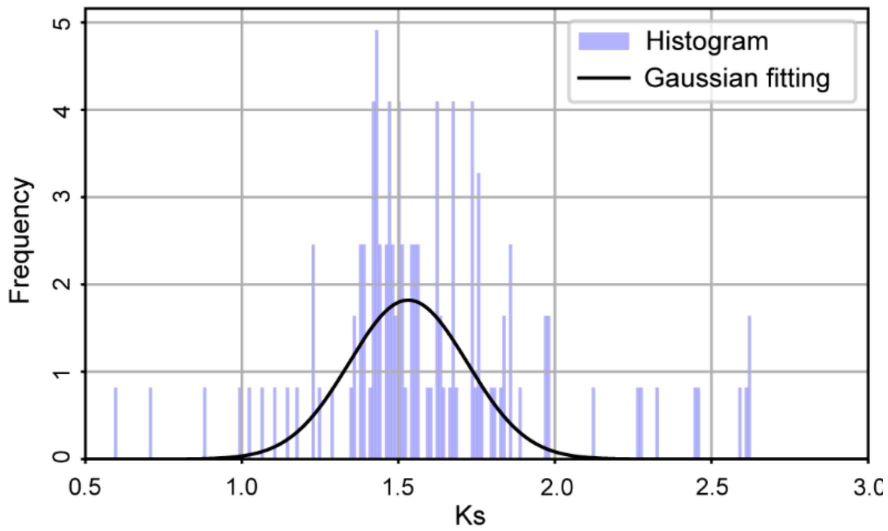

Supplementary Fig. 6. Gaussian fitting of K_s distribution. Gaussian fitting of the K_s distribution in the *B. insignis* genome, showing a peak around K_s = 1.7, which represents the signature of a whole-genome duplication (WGD) event.

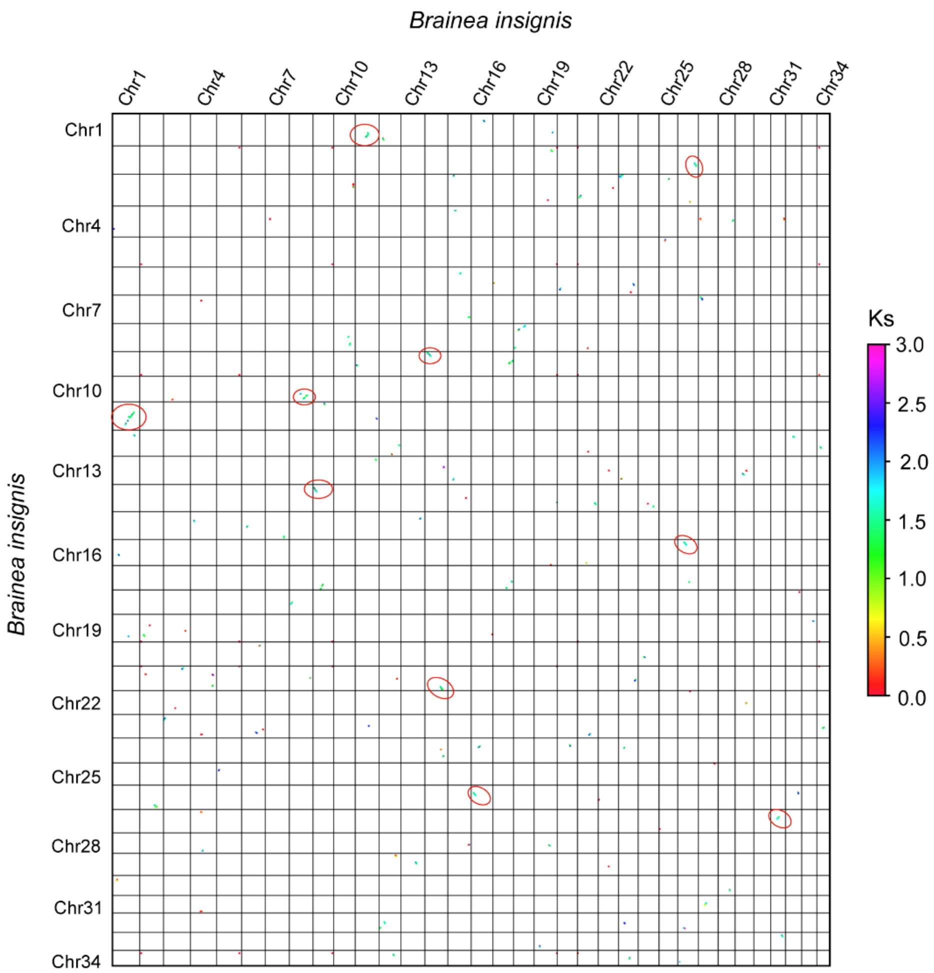

Supplementary Fig. 7. Syntenic blocks within the genome of *B. insignis*. Different colored dots represent the syntenic regions with varying K_s values. The red circles highlight syntenic blocks derived from the WGD event, which are shared with core leptosporangiate ferns.

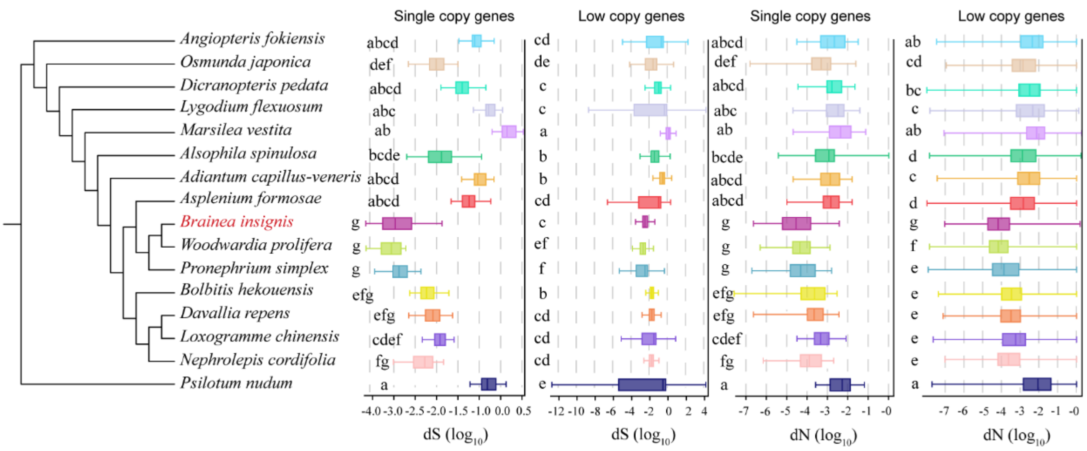

Supplementary Fig. 8. Comparative analysis of evolutionary rates across 16 fern species. The phylogenetic tree was constructed based on 31 single-copy genes, with dS and dN values for 8,720 low-copy genes and 31 single-copy genes displayed on the right. Statistical significances among different species are indicated by letters (a, b, c, etc.) above each bar, where similar letters denote no significant difference and different letters indicate significant variation. Notably, *B. insignis* is highlighted in red within the phylogenetic tree. Source data are provided as a Source Data.

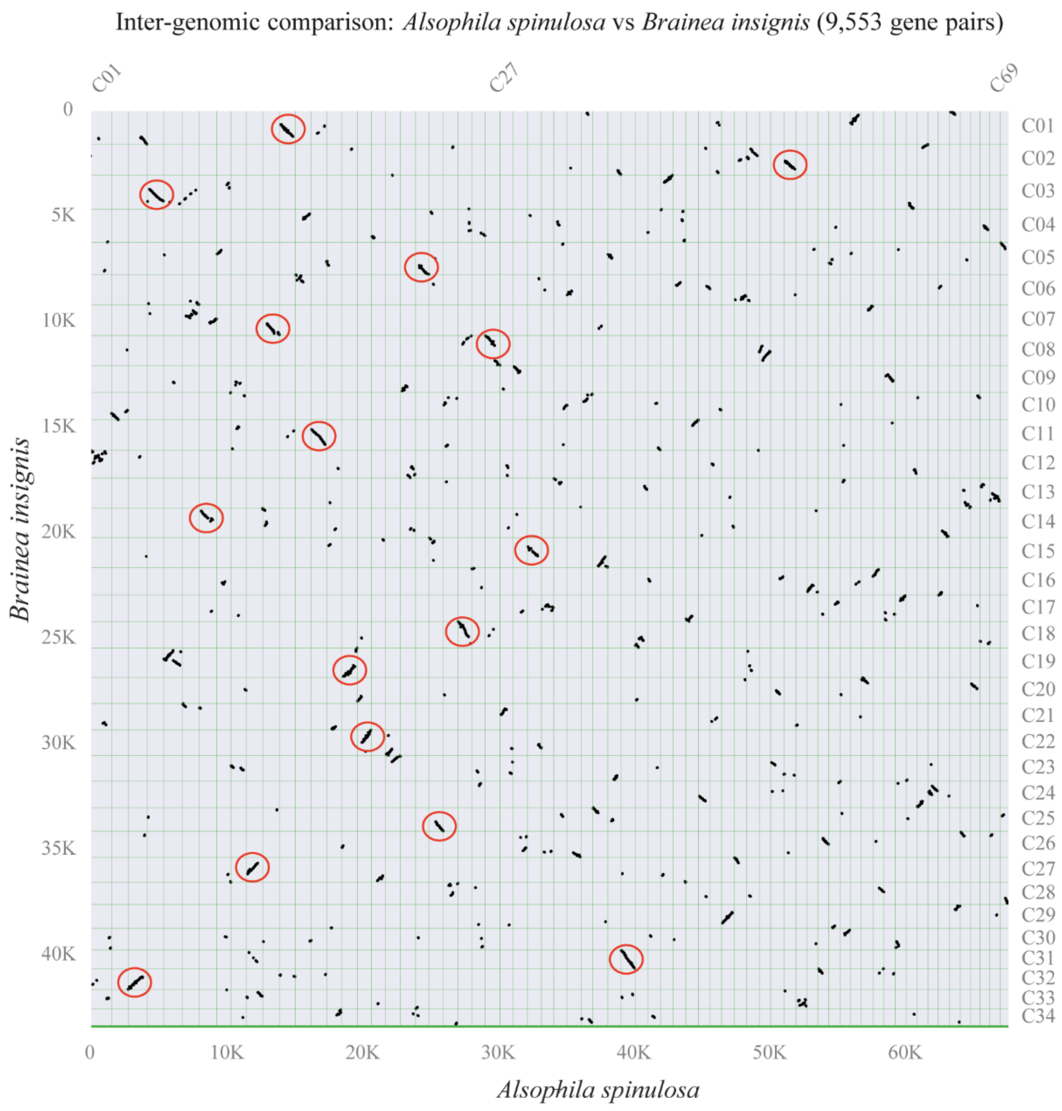

Supplementary Fig. 9. Synteny analysis between *Alsophila spinulosa* and *Brainea insignis*.

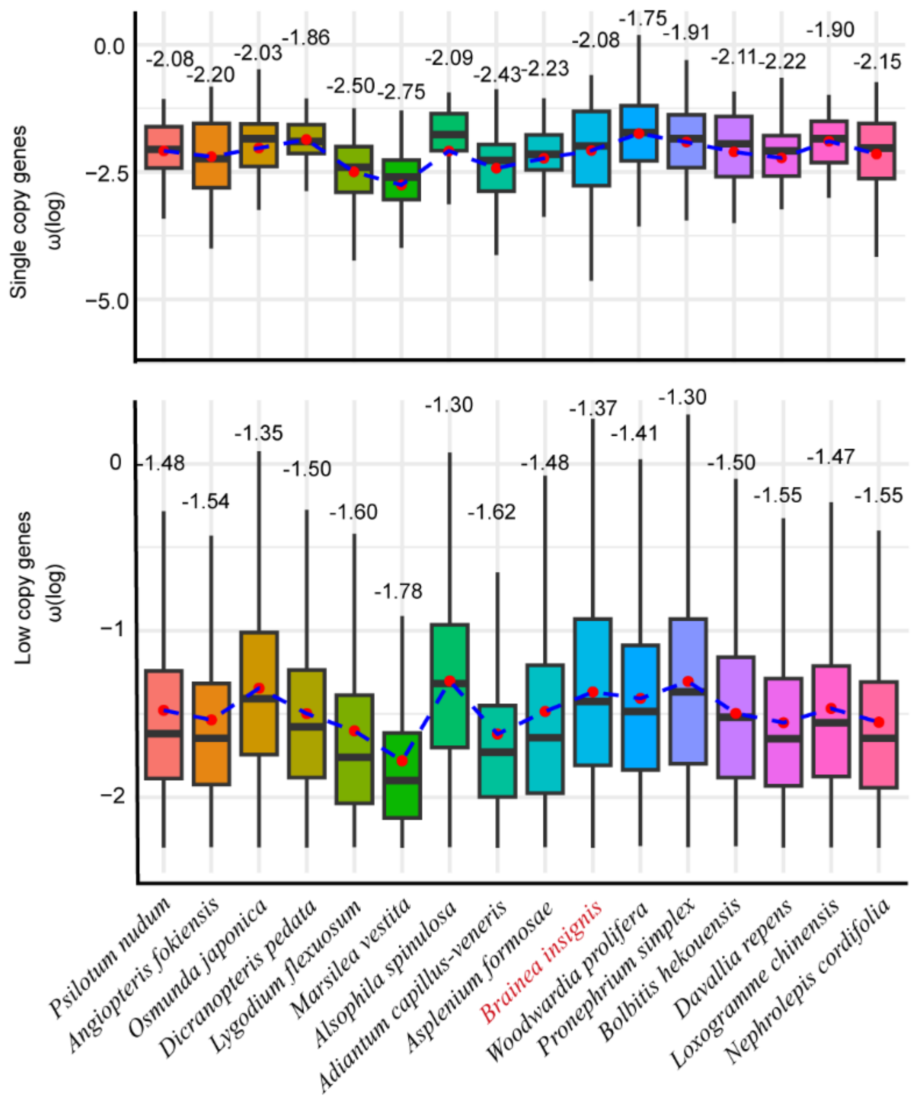

Supplementary Fig. 10. Comparison of ω (dN/dS) values for single-copy and low-copy genes across 16 fern species. Boxplot comparison of ω (dN/dS) values for single-copy and low-copy genes across 16 fern species. Red dots represent the mean values, connected by a blue dashed line, with the mean values displayed above the plots.

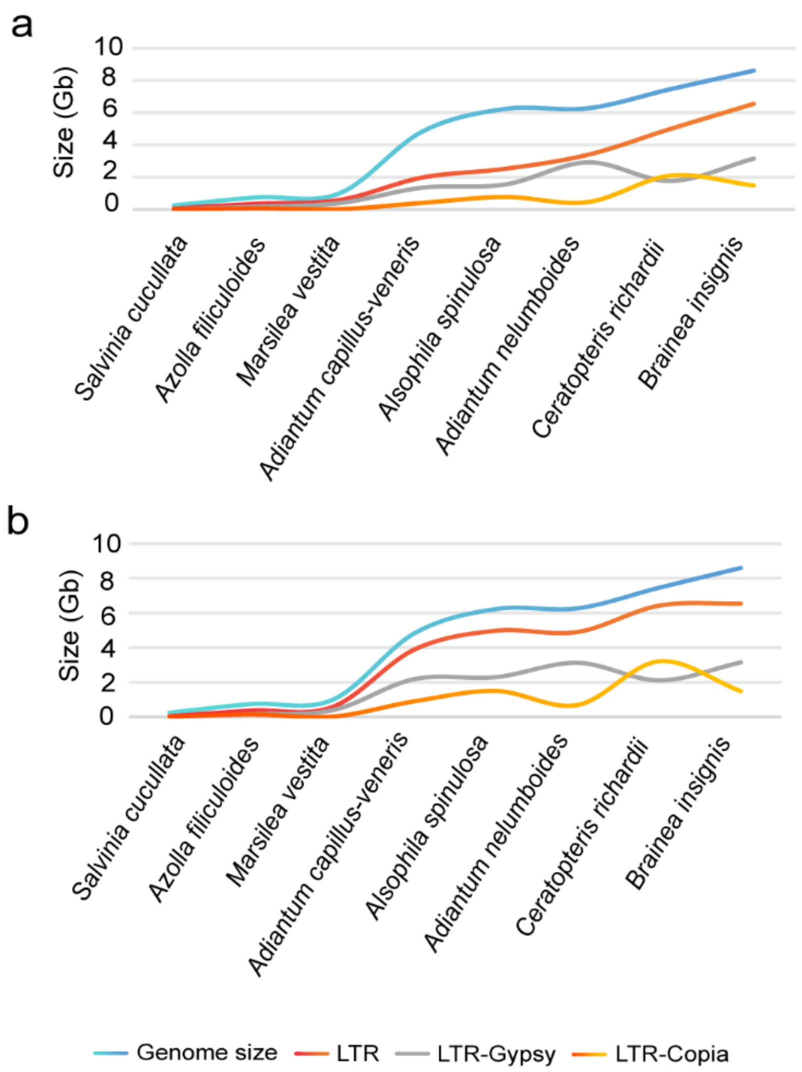

Supplementary Fig. 11. Genome size dynamics in eight fern species. Curve plots showing the genome size and LTR size distribution in 8 fern species, with separate curves for the LTR-Gypsy and LTR-Copia families. **a,** LTR size distributions (including the relative proportions of LTR-Gypsy and LTR-Copia) derived from data reported in the original publications. **b,** LTR size distributions and the relative proportions of LTR-Gypsy and LTR-Copia identified using a unified annotation pipeline in this study.

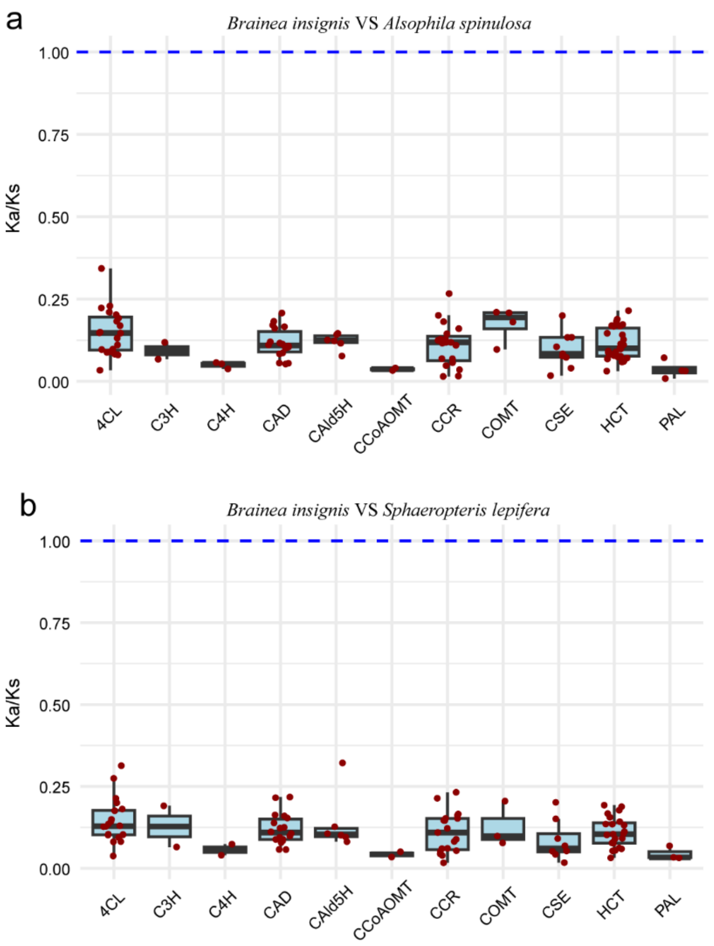

Supplementary Fig. 12. Selection pressure on 11 lignin metabolism-related gene families in tree ferns. The figure shows the selection pressure on 11 gene families related to lignin metabolism. **a**, Positive selection analysis of 1:1 orthologous gene pair between *B. insignis* and *Alsophila spinulosa*. **b**, Positive selection analysis of 1:1 orthologous gene pair between *B. insignis* and *Sphaeropteris lepifera*.

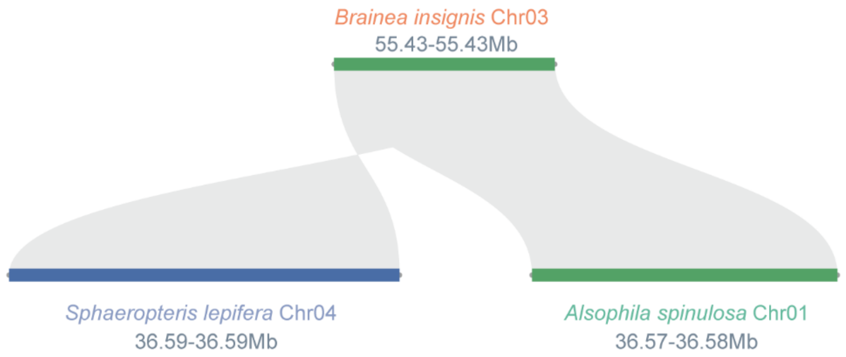

Supplementary Fig. 13. Microsynteny analysis of lignin-related genes across three fern species.

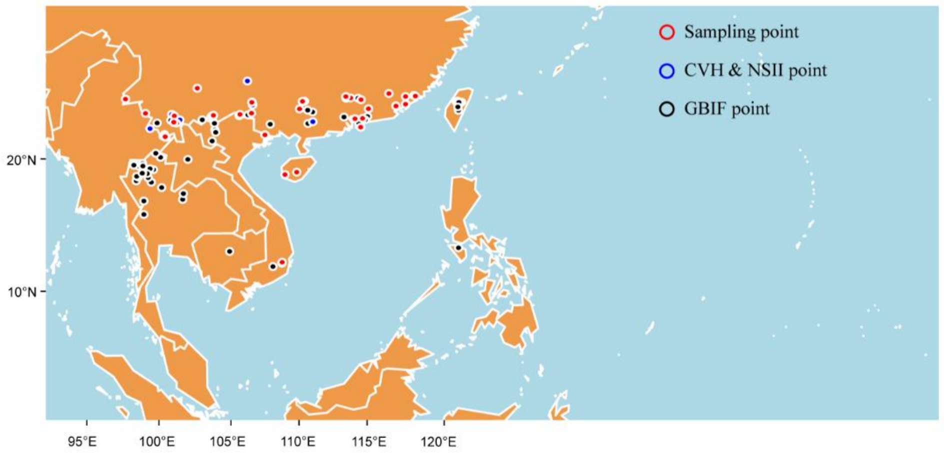

Supplementary Fig. 14. A total of 178 non-redundant occurrence records of *Brainea insignis*. Red, blue, and black points represent sampling locations from this study, occurrence records from NSII (National Specimen Information Infrastructure, China) and CVH (Chinese Virtual Herbarium), and GBIF (Global Biodiversity Information Facility) records, respectively.

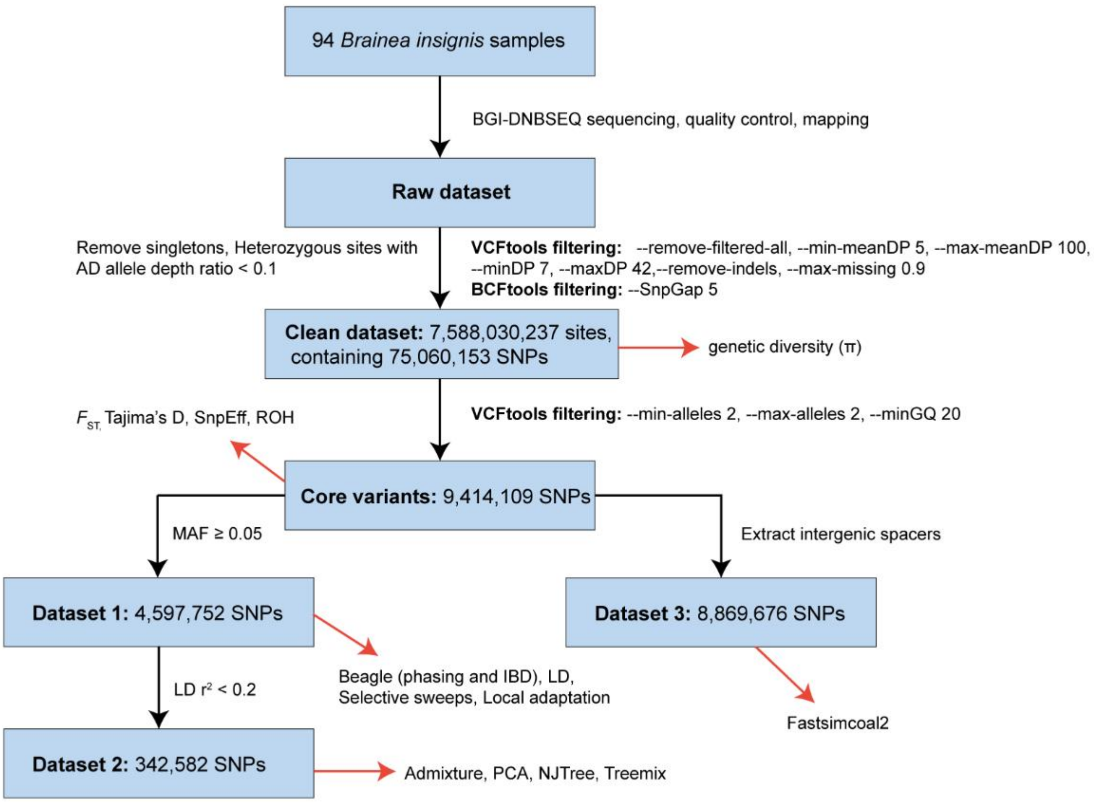

Supplementary Fig. 15. Workflow for SNP filtering in population genomic analysis. This diagram illustrates the workflow for SNP filtering in population genomic analysis. The red arrows indicate the analyses for which the dataset is used.

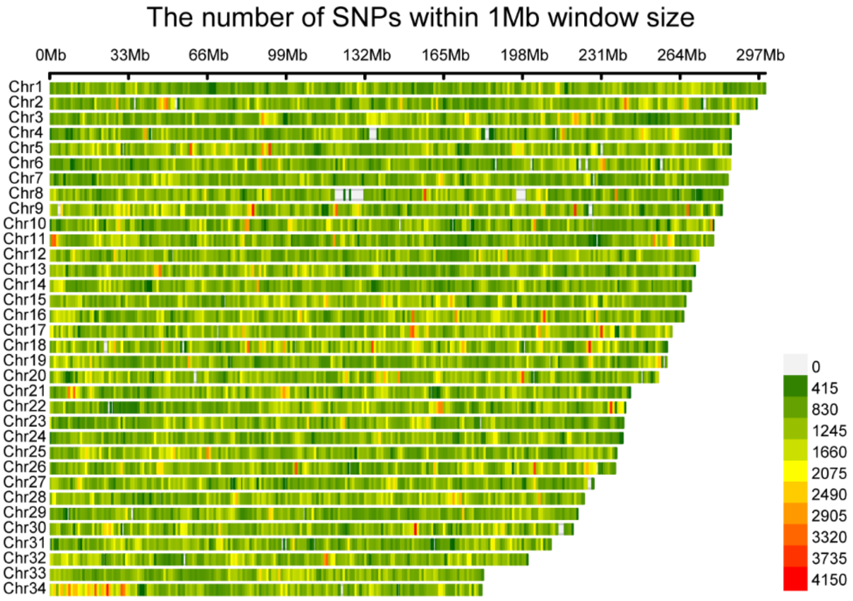

Supplementary Fig. 16. SNP (core variants) density distribution across chromosomes in 1Mb windows.

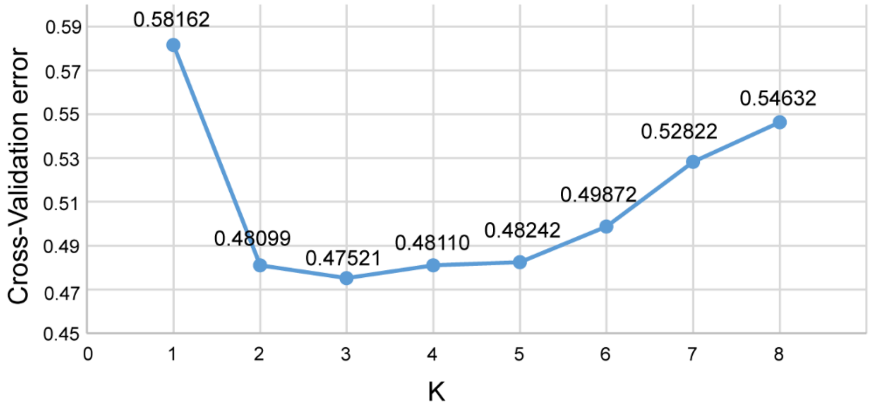

Supplementary Fig. 17. Cross-validation error curve based on neutral loci in Admixture.

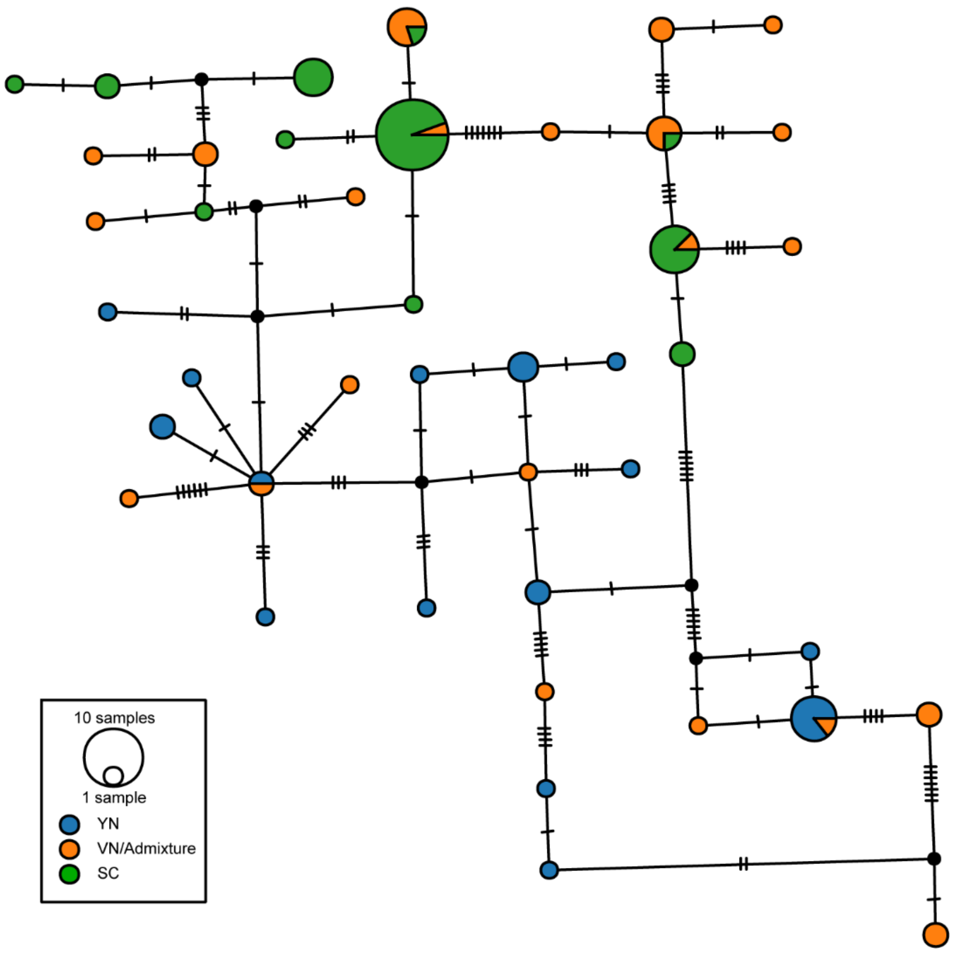

Supplementary Fig. 18. Chloroplast haplotype network. Haplotype network of chloroplast genomes for three lineages, where the size of each circle represents the number of samples associated with each haplotype. The network illustrates the relationships and distribution of chloroplast haplotypes across the lineages.

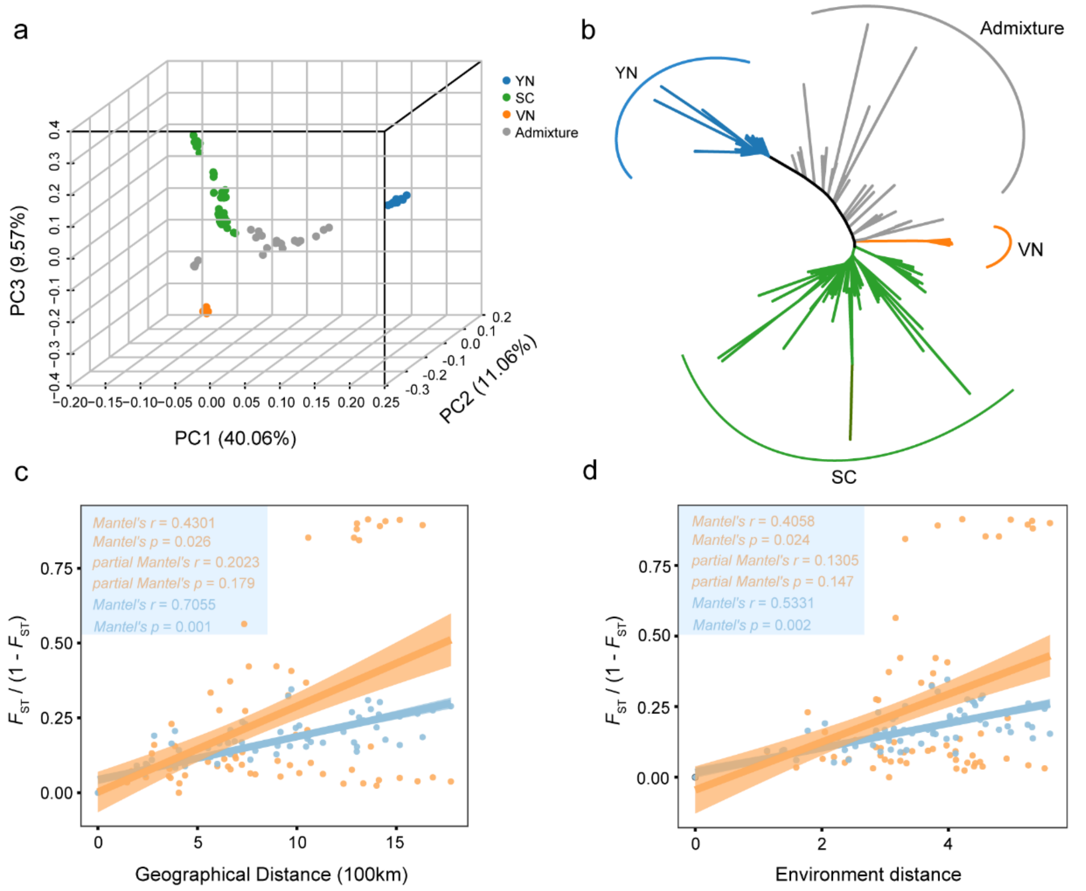

Supplementary Fig. 19. Comprehensive analysis of genetic structure across populations. a, 3D PCA plot visualizes the genetic variation along the first three principal components (PC1, PC2, and PC3), revealing distinct clustering of populations based on genetic similarity. Each point represents a population, with colors indicating different groups. **b,** NJ tree illustrating the phylogenetic relationships among different lineages, with branches color-coded to represent distinct groups. **c,** IBD analysis (Mantel test, two-sided) showing the relationship between geographic distance and genetic distance for populations, separately for neutral variants (blue dots and line) and adaptive variants (orange dots and line). **d,** IBE analysis (Mantel test, two-sided) showing the relationship between environmental distance and genetic distance for populations, separately for neutral variants (blue dots and line) and adaptive variants (orange dots and line).

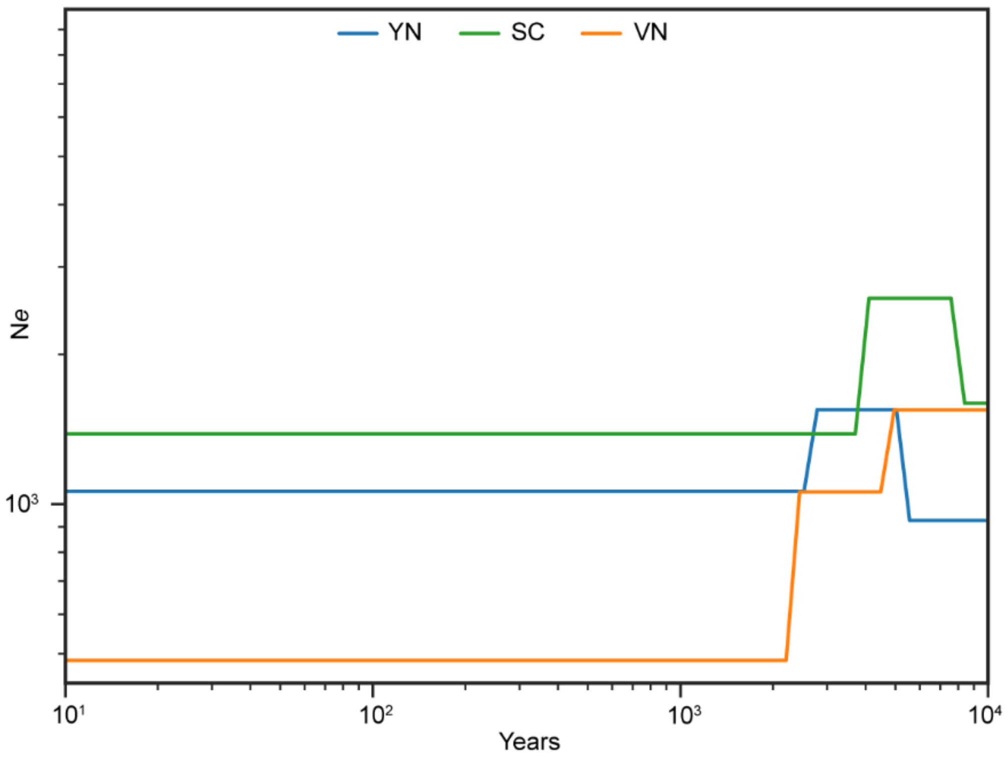

Supplementary Fig. 20. Population fluctuations in demographic history estimated by SMC++. The figure shows the demographic history of three lineages, represented by different colors in the legend. This analysis estimates the population dynamics for each individual lineage.

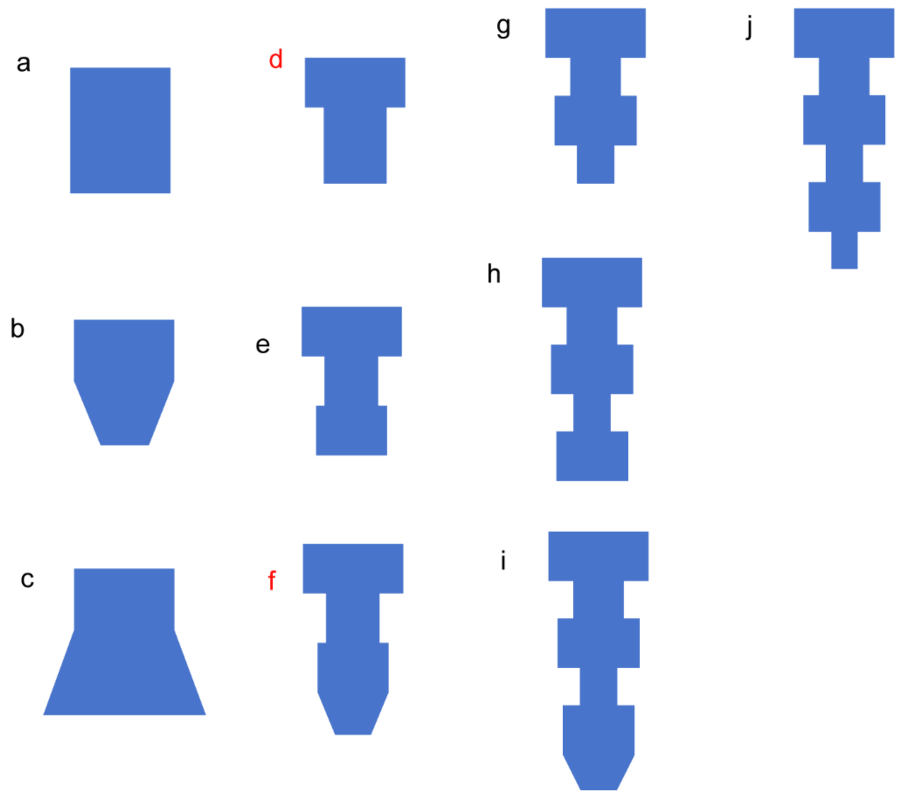

Supplementary Fig. 21. Ten kinds of demography models. The red numbers mark the selected best model. (a) Population size is constant. (b) Population experiences a contraction. (c) Population experiences an expansion. (d) Population undergoes one bottleneck event. (e) Population undergoes one bottleneck event followed by expansion. (f) Population undergoes one bottleneck event followed by contraction. (g) Population undergoes two bottleneck events. (h) Population undergoes two bottleneck events followed by expansion. (i) Population undergoes two bottleneck events followed by contraction. (j) Population undergoes three bottleneck events.

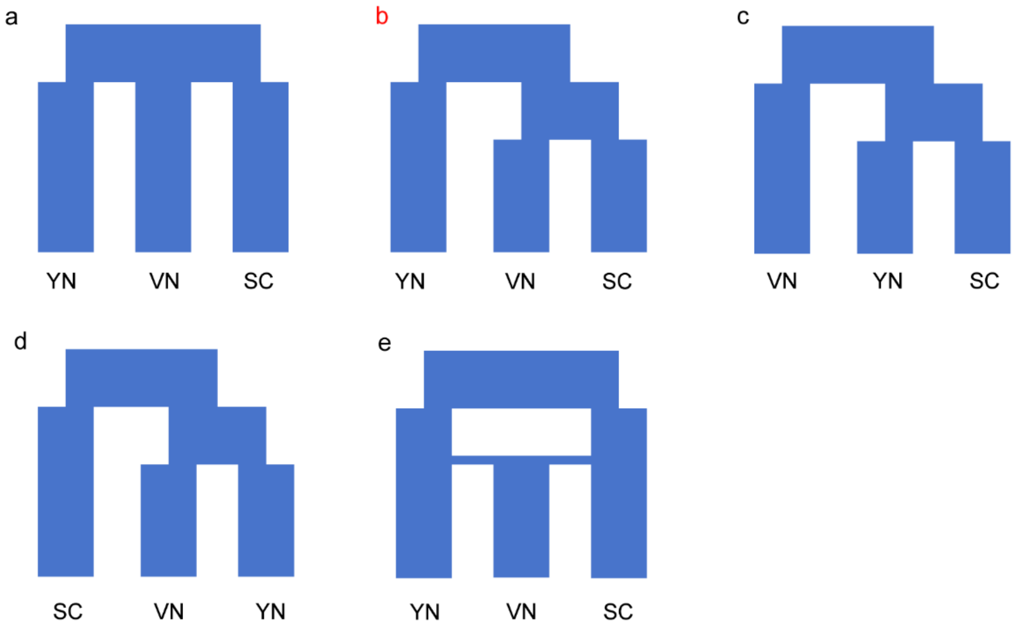

Supplementary Fig. 22. Five kinds of demography differentiation models. The red numbers mark the selected best model. (a) The divergence times of the three lineages are the same. (b) The YN lineage diverged first. (c) The VN lineage diverged first. (d) The SC lineage diverged first. (e) The VN lineage is a mixture of the YN and SC lineages.

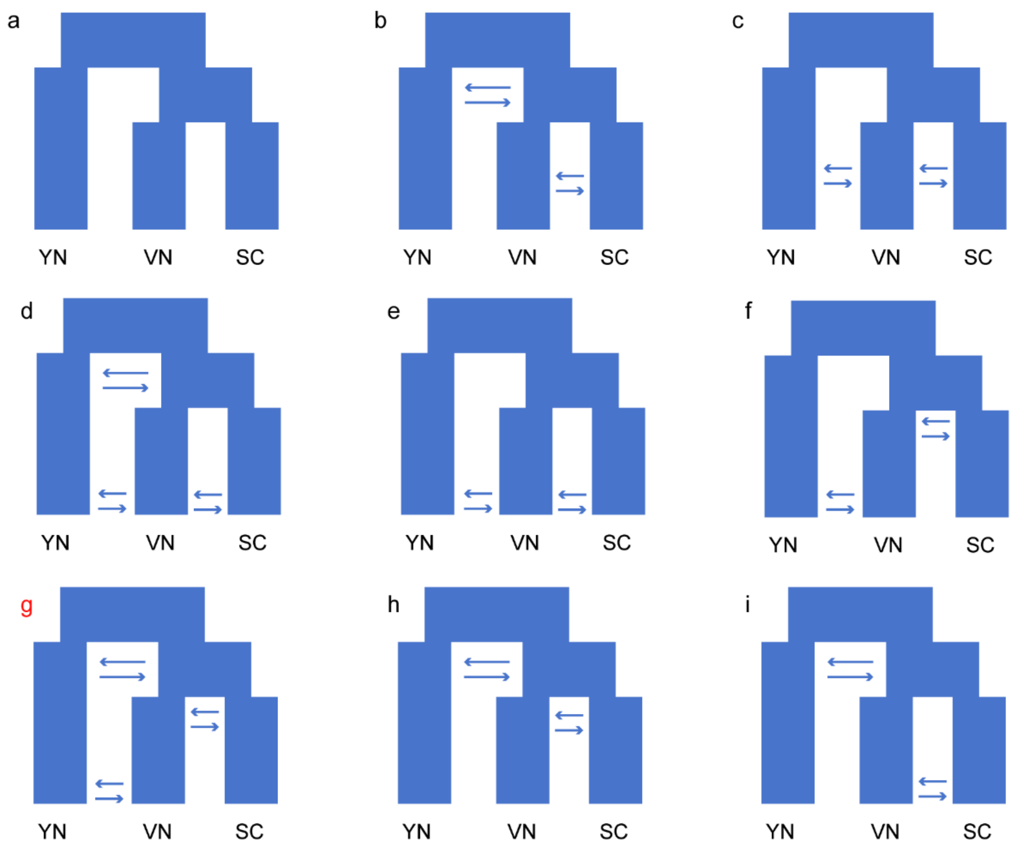

Supplementary Fig. 23. Nine kinds of models for estimation of the process of differentiation when different gene flow scenarios are considered. The red numbers mark the selected best model. (a) No gene flow occurs between the lineages. (b) Ancient gene flow occurred between the YN lineage and the common ancestor of VN and SC lineages, and there is ongoing gene flow between the VN and SC lineages. (c) Ongoing gene flow exists between the VN lineage and both the YN and SC lineages. (d) Ancient gene flow occurred between the YN lineage and the common ancestor of VN and SC lineages, and recent gene flow exists between the VN lineage and both the YN and SC lineages. (e) Recent gene flow exists between the VN lineage and both the YN and SC lineages. (f) Recent gene flow exists between the VN and YN lineages, and ancient gene flow occurred between the VN and SC lineages. (g) Ancient gene flow occurred between the YN lineage and the common ancestor of VN and SC lineages. Recent gene flow exists between the VN and YN lineages, and ancient gene flow occurred between the VN and SC lineages. (h) Ancient gene flow occurred between the YN lineage and the common ancestor of VN and SC lineages, and ancient gene flow occurred between the VN and SC lineages. (i) Ancient gene flow occurred between the YN lineage and the common ancestor of VN and SC lineages, and recent gene flow exists between the VN and SC lineages.

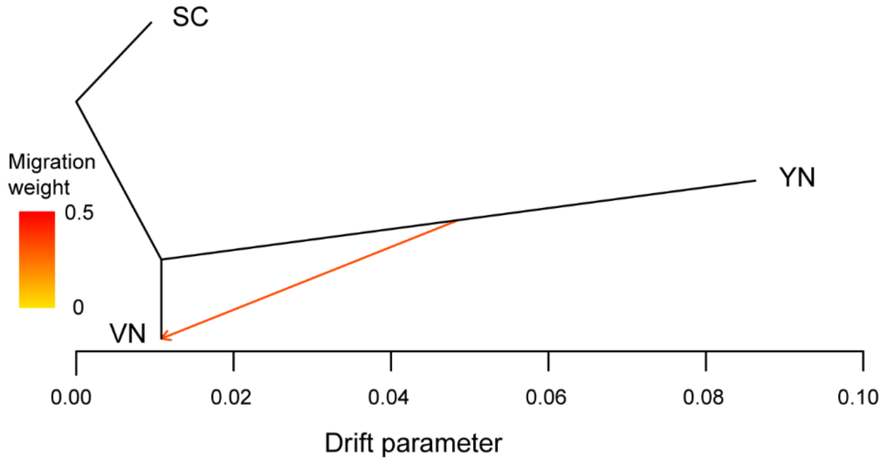

Supplementary Fig. 24. TreeMix trees with 1 migration events from lineage YN to VN.

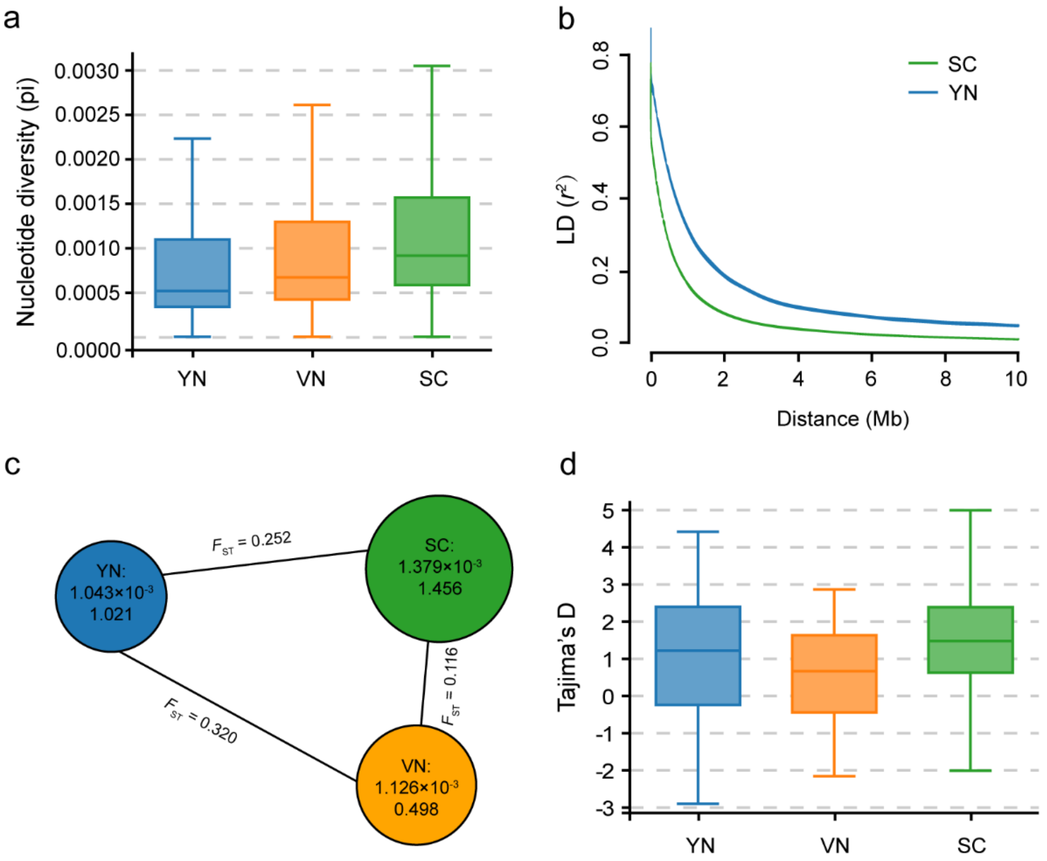

Supplementary Fig. 25. Comprehensive analysis of genetic diversity among lineages. a, Boxplot showing the distribution of nucleotide diversity (π) across individuals from three different lineages. The plot illustrates the median, interquartile range, and outliers of π for each lineage, highlighting differences in genetic diversity among the lineages. **b**, LD decay plot showing the relationship between genetic distance and linkage disequilibrium (LD) across different lineages. The plot illustrates how LD decreases with increasing genetic distance, with distinct patterns of decay observed for each lineage. **c**, Estimates of nucleotide genetic diversity (average π, indicated inside the circle) and Wright's fixation index (F_{ST}, shown on the solid line) for the three lineages, with circle size corresponding to the average π value. **d**, Boxplot showing the distribution of Tajima's *D* values across the three lineages, with each box representing the range, median, and outliers of Tajima's D for each lineage.

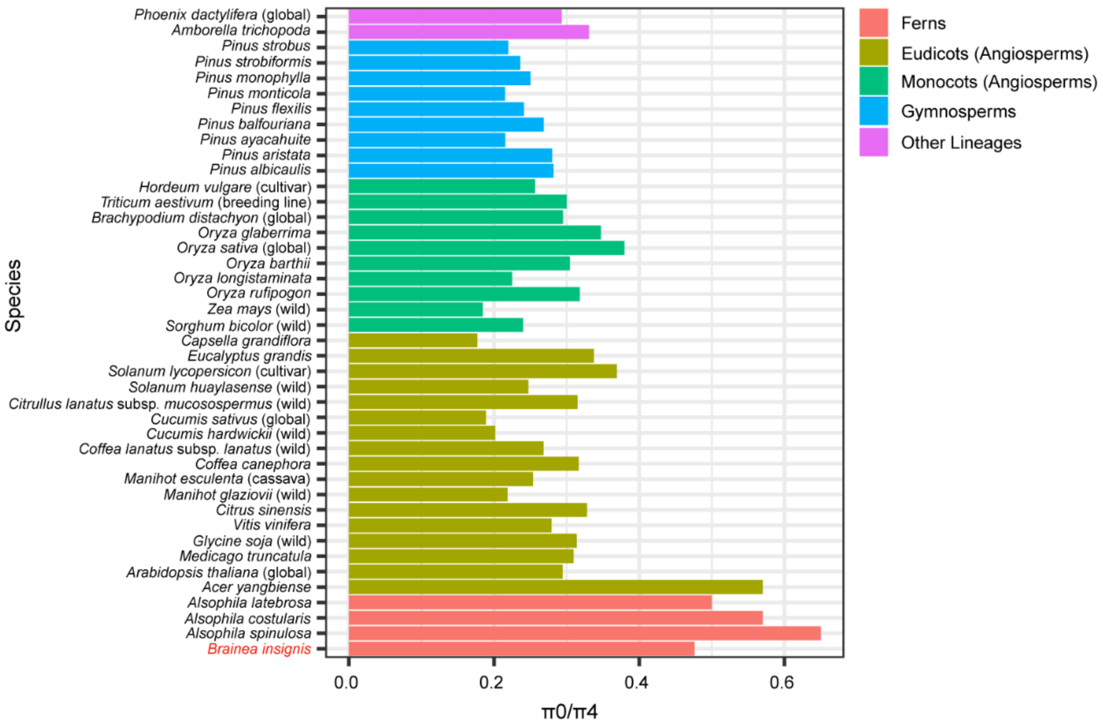

Supplementary Fig. 26. Summary of π_0/π_4 ratios across fern and seed plant species.

Data were obtained from References [25], [26] and [27]. The *Brainea insignis* is marked in red.

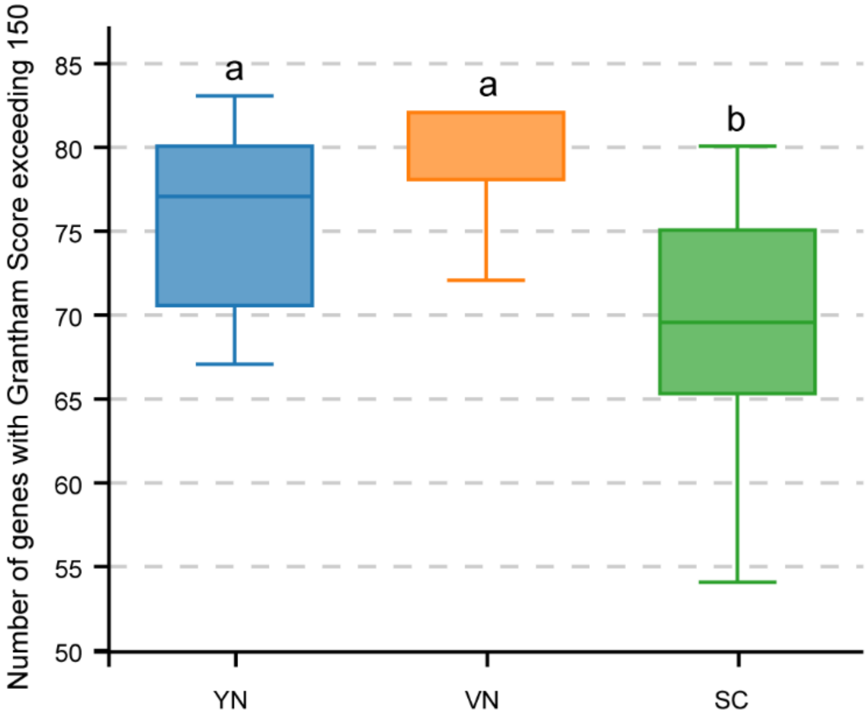

Supplementary Fig. 27. Genetic load estimated by Grantham Score (GS). The derived deleterious missense variants were predicted by GS (≥150). The box plots indicate the distribution of GS, with different letters (a, b) denoting significant differences between lineages.

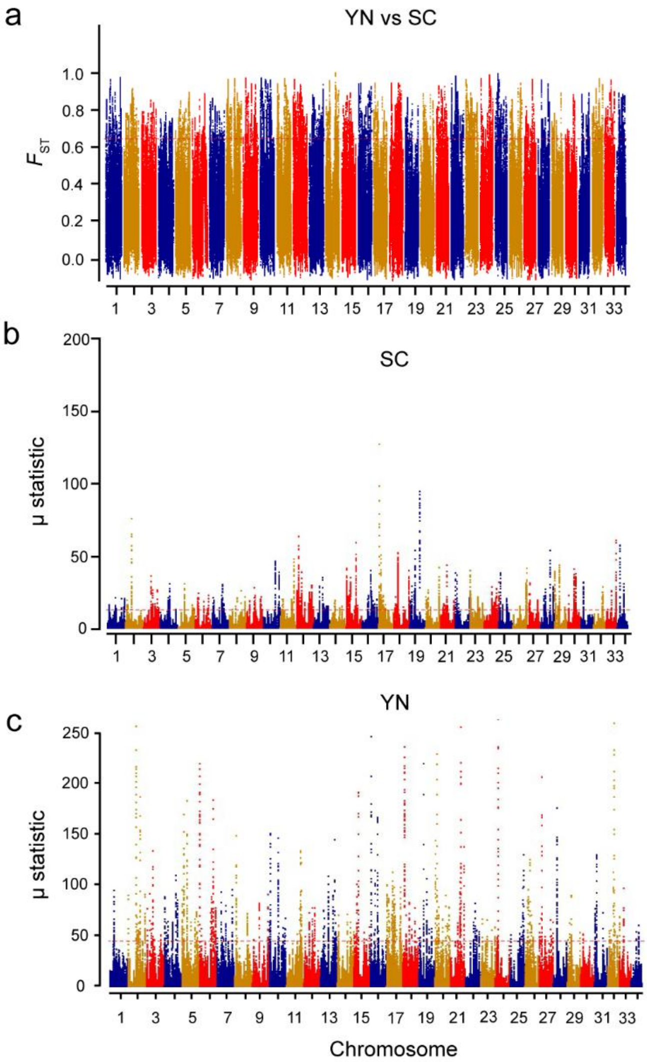

Supplementary Fig. 28. Selective sweep analyses of YN and SC lineages. a, *F*st sliding window analysis between YN and SC lineages, with the horizontal red dashed line representing the top 5% threshold. **b**, Whole-genome screening of the SC lineage using RAiSD (Raised Accuracy in Sweep Detection), where the red dashed line represents μ statistic values above the 99.99% quantile. **c**, Whole-genome screening of the YN lineage using RAiSD, with the red dashed line also representing μ statistic values above the 99.99% quantile.

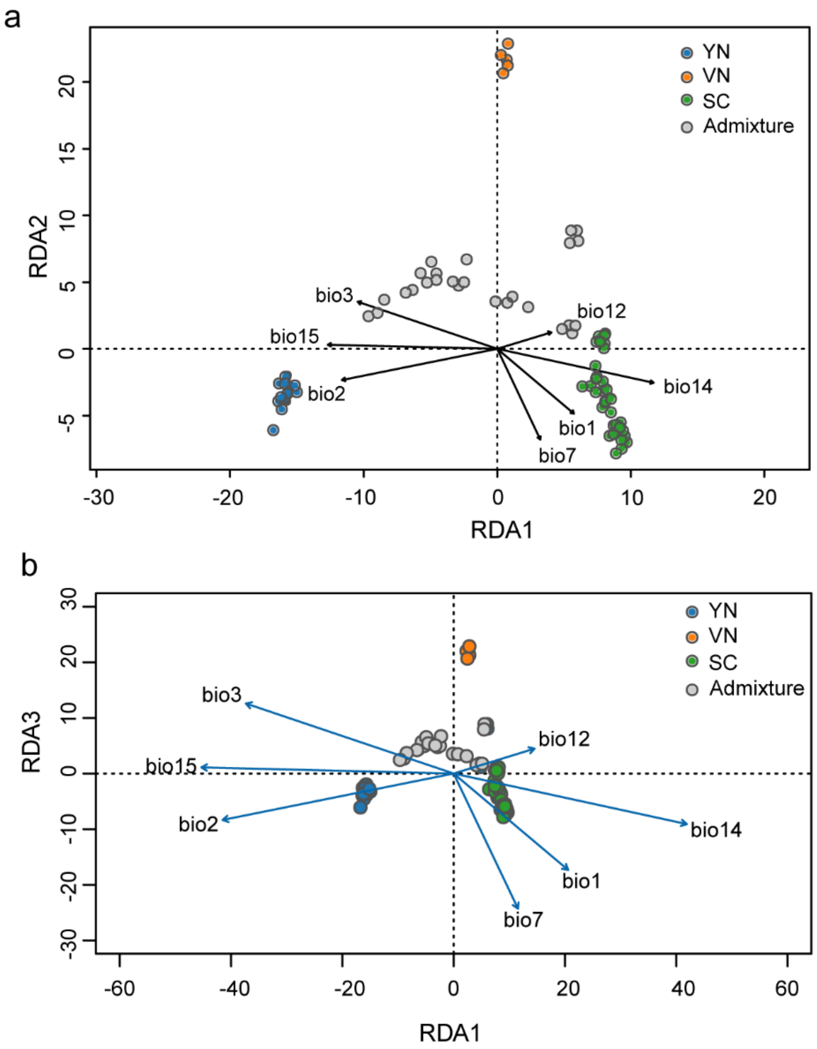

Supplementary Fig. 29. RDA analysis showing population adaptive differentiation. **a**, RDA analysis of local adaptation loci and climate factors, showing the relationship between RDA1 and RDA2, highlighting adaptive differentiation between lineages. **b**, RDA analysis between local adaptation loci and climate factors, showing the relationship between RDA1 and RDA3, further illustrating the adaptive divergence among the lineages.

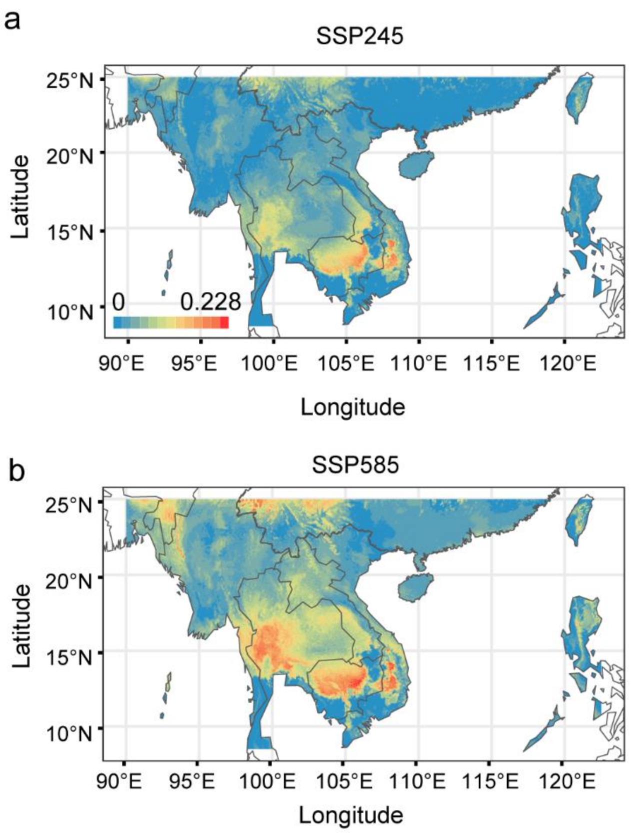

Supplementary Fig. 30. Simulated local offsets for the period 2081-2100 under different SSP scenarios. a, Simulated local offset for the period 2081-2100 under the SSP245 scenario. **b**, Simulated local offset for the period 2081-2100 under the SSP585 scenario.

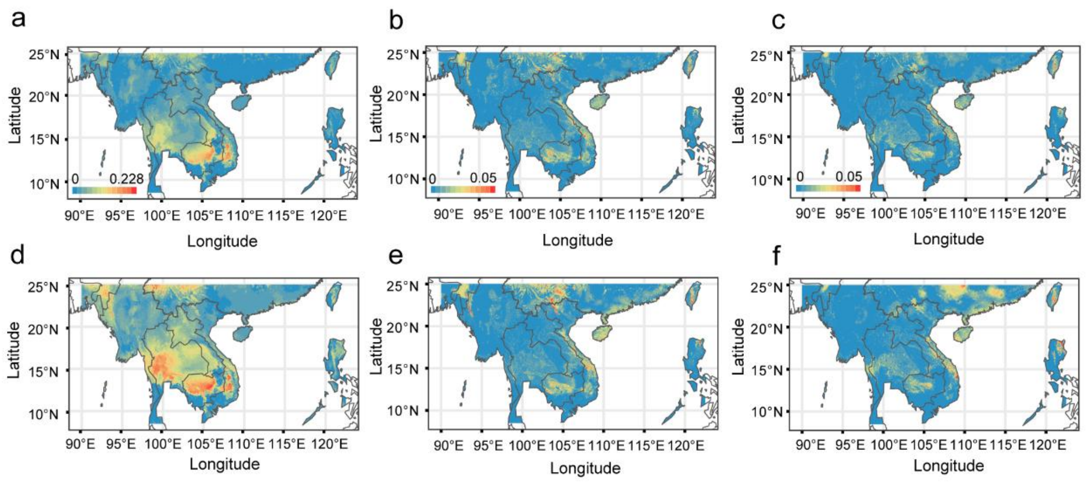

Supplementary Fig. 31. Patterns of genetic offset under different climate change scenarios. Panels a, b, c show results under the SSP245 (moderate) scenario, while panels d, e, f correspond to the SSP585 (extreme) scenario. Local offset (a, d); Forward offset (b, e); Reverse offset (c, f).

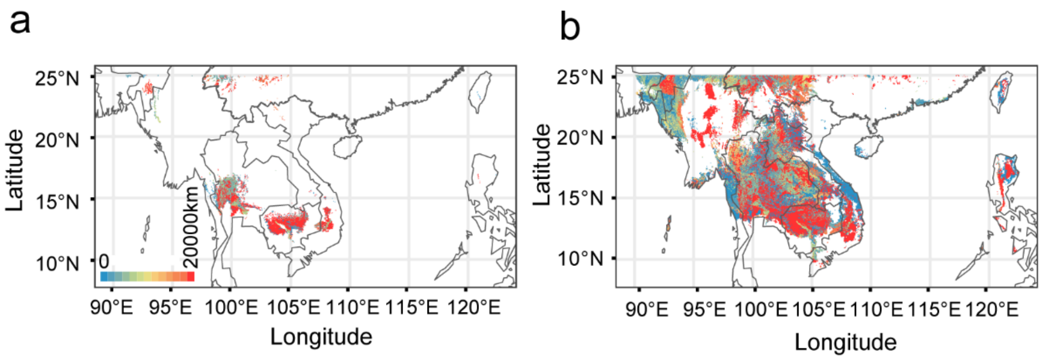

Supplementary Fig. 32. Population resilience and required migration distances under different genetic offset thresholds in *B. insignis*. a, High threshold (>0.15): most populations are locally adapted; only a few in the Indochinese Peninsula require long-distance migration. **b**, Moderate threshold (>0.05): required migration distances increase dramatically, with many populations exceeding 5,000 km.

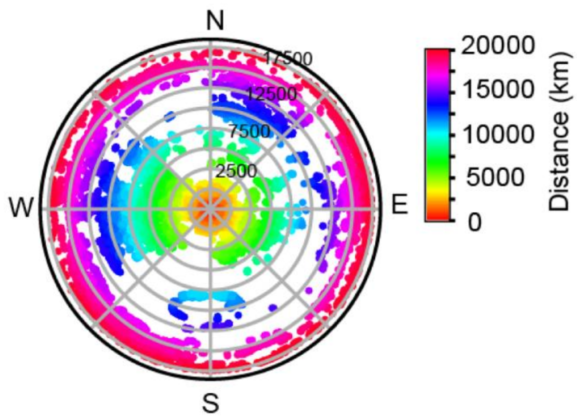

Supplementary Fig. 33. Polar plot showing forward offset migration direction and distance.

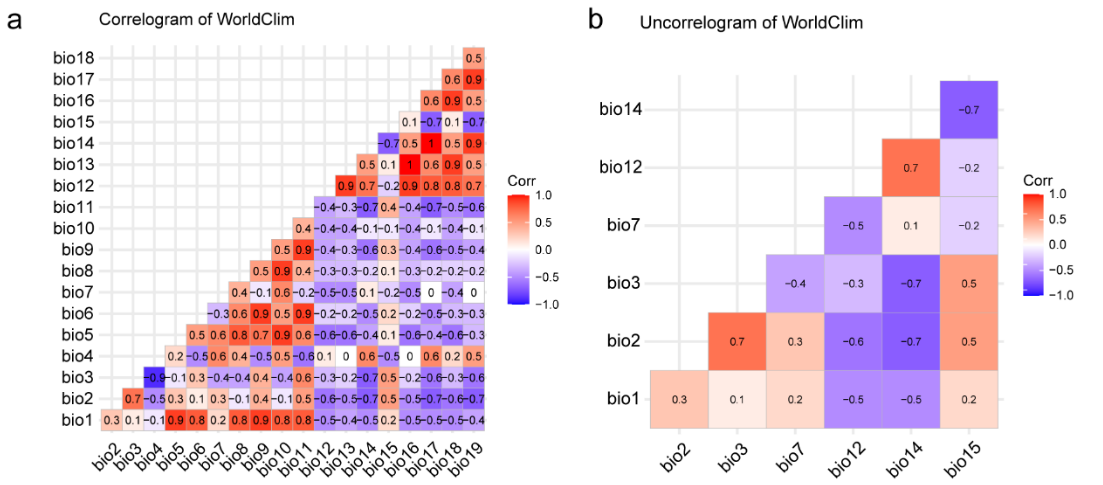

Supplementary Fig. 34. Correlation analysis of climate factors. a, Heatmap showing the correlation between 19 climate factors, illustrating the strength and direction of their relationships. **b**, Heatmap showing the correlation between 6 climate factors with correlation coefficients below 0.7, highlighting weaker relationships among these factors.

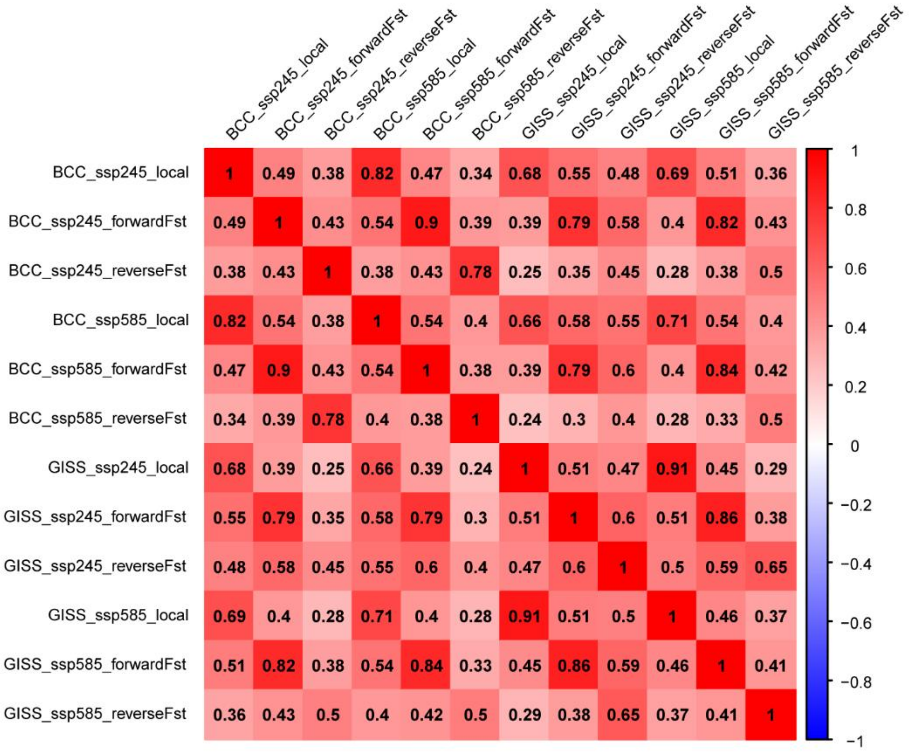

Supplementary Fig. 35. Correlation heatmap of genetic offsets simulated based on two climate models (BCC and GISS). The plot illustrates the relationship between genetic offsets predicted by these models, highlighting similarities in their simulations.

Supplementary Table 1. A summary of raw and clean data volumes for different sequencing technologies.

Library type	Raw data (Gb)	Clean data (Gb)
Illumina	512.37	510.90
PacBio HiFi	-	359.99
Hi-C	1,404.44	1,393.88
RNA-seq	46.79	45.64

Supplementary Table 2. Statistical summary of Hi-C sequencing data for five libraries.

Library name	Total reads pairs	Unique di-tags	Effect rate	Average effect rate
FDHC230003598-1a_L1	5,945,912	1,608,570	27.05%	26.47%
FDHC230003598-2a_L4	7,347,333	2,362,108	25.54%	
FDHC230003598-3a_L5	6,640,837	1,748,540	26.33%	
FDHC230003598-4a_L3	7,396,106	2,002,876	27.08%	
FDHC230003598-5a_L6	7,135,115	1,883,130	26.39%	

Supplementary Table 3. BUSCO assessment for both assembly and annotation.

**Assembly assessment**		
	**Number**	**Percentage (%)**
Complete BUSCOs	801	97.4
Complete and single-copy BUSCOs	739	89.9
Complete and Duplicated BUSCOs	62	7.5
Fragmented BUSCOs	17	2.1
Missing BUSCOs	4	0.5
Total BUSCOs groups searched	822	-
**Annotation assessment**		
	**Number**	**Percentage (%)**
Complete BUSCOs	692	84.2
Complete and single-copy BUSCOs	650	79.1
Complete and Duplicated BUSCOs	42	5.1
Fragmented BUSCOs	104	12.7
Missing BUSCOs	26	3.2
Total BUSCOs groups searched	822	-

Supplementary Table 4. Repeat sequence annotation statistics.

Type	Number	Length (bp)	Percentage
SINE	2,147	1,028,719	0.01%
LINE	482,516	502,995,628	5.82%
LTR	2,362,979	4,145,887,371	47.99%
DNA transposons	2,244,986	2,106,921,533	24.39%
Unknown	241,079	241,869,758	2.80%
Total	-	7,064,135,330	81.77%

Supplementary Table 5. Genomic structural annotation statistics.

Functional annotation type	Number
Gene number	43,573
Average gene length (bp)	15,096.24
Average CDS length (bp)	1,084.84
Average exons per gene	3.84
Average exon length (bp)	282.76
Average intron length (bp)	4,939.39

Supplementary Table 6. Functional annotation of genes across different databases.

Functional annotation database	Number	Percent
NR	35,392	81.22%
Swissport	24,341	55.86%
KEGG	25,347	58.17%
InterPro	37,079	85.10%
Pfam	26,427	60.65%
GO	20,031	45.97%
Annotated	39,118	89.78%

Supplementary Table 7. Relative rate testes of *B. insignis* versus the other ferns using the 31 single copy genes and *P. nudum* as the outgroup.

Ingroup1	Ingroup2	Outgroup	Genes	Identical	Divergent	Ingroup1 specific	Ingroup2 specific	Outgroup specific	Slow	χ^2	*P*-value
A. fokiensis	*B. insignis*	*P. nudum*	31	7541	1027	633	1187	952	*A. fokiensis*	168.64	1.00E-05
O. japonica	*B. insignis*	*P. nudum*	31	8557	1031	477	1069	1301	*O. japonica*	226.69	1.00E-05
D. pedata	*B. insignis*	*P. nudum*	31	7743	969	541	835	1267	*D. pedata*	62.82	1.00E-05
L. flexuosum	*B. insignis*	*P. nudum*	31	8244	1014	580	758	1450	*L. flexuosum*	23.68	1.00E-05
M. vestita	*B. insignis*	*P. nudum*	31	8222	1098	778	648	1625	*B. insignis*	11.85	5.80E-04
A. spinulosa	*B. insignis*	*P. nudum*	31	8330	791	494	518	1948	-	0.57	4.51E-01
A. capillus-veneris	*B. insignis*	*P. nudum*	31	8451	763	499	382	2175	*B. insignis*	15.54	8.00E-05
A. formosae	*B. insignis*	*P. nudum*	31	8378	671	364	308	2332	*B. insignis*	4.67	3.08E-02
W. prolifera	*B. insignis*	*P. nudum*	31	8979	264	156	109	3033	*B. insignis*	8.34	3.89E-03
P. simplex	*B. insignis*	*P. nudum*	31	8748	355	166	201	2847	-	3.34	6.77E-02
B. hekouensis	*B. insignis*	*P. nudum*	31	7893	474	254	238	2392	*B. insignis*	0.52	4.71E-01
D. repens	*B. insignis*	*P. nudum*	31	8082	555	286	280	2321	*B. insignis*	0.06	8.00E-01
L. chinensis	*B. insignis*	*P. nudum*	31	7186	547	345	302	1959	-	2.86	9.09E-02
N. cordifolia	*B. insignis*	*P. nudum*	31	8321	473	216	252	2461	-	2.77	9.61E-02

Note: The ‘slow’ column identifies ingroup species that have evolved significantly more slowly, based on *P*-values. Species that do not show significant differences are indicated with a ‘-‘. The ‘idential’ and ‘divergent’ columns refer to sites where the amino acid residue is the same or different in all 3 sequences, respectively. ‘Ingroup1-specific’ column refers to sites where ingroup2 and outgroup share the same amino acid but not ingroup 1. The same applies for ‘ingroup2-specific’ and ‘outgroup-specific’. The green background blocks highlight comparisons of evolutionary rates with the core leptosporangiate ferns, while the red background blocks highlight comparisons of evolutionary rates with early-diverging ferns. Statistical significance of relative rate differences was determined using a one-sided chi-square (χ^2) test.

Supplementary Table 8. AIC evaluation of demographic models (Supplementary Fig. 21).

Model	Statistics	YN	VN	SC
Demography-a	AIC	43248904.513	44528667.907	67526074.612
	ABS(Δlhood)	5060878.812	7995581.368	6691925.040
Demography-b	AIC	43143956.741	44496670.180	67525724.282
	ABS(Δlhood)	5038088.390	7988631.847	6691847.664
Demography-c	AIC	43221226.758	44496670.180	67132291.823
	ABS(Δlhood)	5054867.361	7988631.847	6606414.891
Demography-d	AIC	43209650.137	44496668.180	67134131.900
	ABS(Δlhood)	5052353.964	7988631.847	6606814.893
Demography-e	AIC	43222128.383	44496672.184	67136780.603
	ABS(Δlhood)	5055062.712	7988631.848	6607389.183
Demography-f	AIC	43120218.195	44496678.180	67092065.215
	ABS(Δlhood)	5032931.893	7988631.847	6597678.057
Demography-g	AIC	43120830.764	44496676.180	67093076.288
	ABS(Δlhood)	5033065.345	7988631.847	6597898.043
Demography-h	AIC	43124570.349	44496680.180	67098741.438
	ABS(Δlhood)	5033876.517	7988631.847	6599127.346
Demography-i	AIC	43120482.353	44496686.180	67092933.019
	ABS(Δlhood)	5032987.517	7988631.847	6597864.761
Demography-j	AIC	43120490.278	44496686.180	67093128.637
	ABS(Δlhood)	5032989.238	7988631.847	6597907.239

Note: The optimal model is highlighted in red font.

Supplementary Table 9. AIC evaluation of lineage divergence models without gene flow (Supplementary Fig. 22).

Model	AIC	ABS(Δlhood)
Model-a	278636590.565	24909524.772
Model-b	190410472.692	5751466.696
Model-c	190733373.138	5821583.637
Model-d	191995500.604	6095651.134
Model-e	190777791.903	5831228.615

Note: The optimal model is highlighted in red font.

Supplementary Table 10. AIC Evaluation of lineage divergence models with gene flow (Supplementary Fig. 23).

Model	AIC	ABS(Δlhood)
Model-a	190643054.480	5801970.321
Model-b	187724105.411	5168126.847
Model-c	189270577.429	5503938.979
Model-d	187488657.515	5116998.683
Model-e	189299398.211	5510196.898
Model-f	191129241.254	5907542.266
Model-g	187285617.730	5072909.154
Model-h	187720831.357	5167415.461
Model-i	187726154.925	5168571.459

Note: The optimal model is highlighted in red font.

Supplementary Table 11. Evaluation of selection in lineages using π_0/π_4 ratios.

Lineage	π0	π4	Ratio (π0/π4)
YN	0.000142	0.0002823	0.503
VN	0.000275	0.000557	0.494
SC	0.000593	0.00138	0.431

Supplementary references

1. Fang, Y. H. *et al*. The genome of homosporous maidenhair fern sheds light on the euphyllophyte evolution and defences. *Nat. Plants* **8**, 1024-1037 (2022).
2. Marchant, D. B. *et al*. Dynamic genome evolution in a model fern. *Nat. Plants* **8**, 1038-1051 (2022).
3. Huang, X. *et al*. The flying spider-monkey tree fern genome provides insights into fern evolution and arborescence. *Nat. Plants* **8**, 500-512 (2022).
4. Rahmatpour, N. *et al*. Analyses of *Marsilea vestita* genome and transcriptomes do not support widespread intron retention during spermatogenesis. *New Phytol*. **237**, 1490-1494 (2023).
5. Wei, Z. Y. *et al*. Resolving the stasis-dynamism paradox: genome evolution in tree ferns. *Mol. Biol. Evol.* **42**, msaf247 (2025).
6. Camacho, C. *et al*. BLAST+: architecture and applications. *BMC Bioinformatics* **10**, 421 (2009).
7. Birney, E., Clamp, M. & Durbin, R. GeneWise and genomewise. *Genome Res*. **14**, 988-995 (2004).
8. Stanke, M., Keller, O., Gunduz, I., Hayes, A., Waack, S. & Morgenstern, B. AUGUSTUS: *ab initio* prediction of alternative transcripts. *Nucleic Acids Res.* **34**, W435-W439 (2006).
9. Korf, I. Gene finding in novel genomes. *BMC Bioinformatics* **5**, 59 (2004).
10. Kim, D., Langmead, B. & Salzberg, S. L. HISAT: a fast spliced aligner with low memory requirements. *Nat. Methods* **12**, 357-360 (2015).
11. Pertea, M., Pertea, G. M., Antonescu, C. M., Chang, T. C., Mendell, J. T. & Salzberg, S. L. StringTie enables improved reconstruction of a transcriptome from RNA-seq reads. *Nat. Biotechnol*. **33**, 290-295 (2015).
12. Haas, B. J. *et al*. Automated eukaryotic gene structure annotation using EVidenceModeler and the program to assemble spliced alignments. *Genome Biol*. **9**, R7 (2008).
13. Griffiths-Jones, S., Bateman, A., Marshall, M., Khanna, A. & Eddy, S. R. Rfam: an RNA family database. *Nucleic Acids Res.* **31**, 439-441 (2003).
14. Nawrocki, E. P. & Eddy, S. R. Infernal 1.1: 100-fold faster RNA homology searches. *Bioinformatics* **29**, 2933-2935 (2013).
15. Lowe, T. M. & Eddy, S. R. tRNAscan-SE: a program for improved detection of transfer RNA genes in genomic sequence. *Nucleic Acids Res*. **25**, 955-964 (1997).
16. Jin, J. J. *et al*. GetOrganelle: a fast and versatile toolkit for accurate *de novo* assembly of organelle genomes. *Genome Biol*. **21**, 241 (2020).
17. Qu, X. J., Moore, M. J., Li, D. Z. & Yi, T. S. PGA: a software package for rapid, accurate, and flexible batch annotation of plastomes. *Plant Methods* **15**, 50 (2019).
18. Kearse, M. *et al*. Geneious Basic: an integrated and extendable desktop software platform

for the organization and analysis of sequence data. *Bioinformatics* **28**, 1647-1649 (2012).
19. Li, H. & Durbin, R. Fast and accurate short read alignment with Burrows–Wheeler transform. *Bioinformatics* **25**, 1754-1760 (2009).
20. Danecek, P. *et al*. Twelve years of SAMtools and BCFtools. *Gigascience* **10**, giab008 (2021).
21. Danecek P. *et al*. The variant call format and VCFtools. *Bioinformatics* **27**, 2156-2158 (2011).
22. Katoh, K., Misawa, K., Kuma, Ki. & Miyata, T. MAFFT: a novel method for rapid multiple sequence alignment based on fast Fourier transform. *Nucleic Acids Res*. **30**, 3059-3066 (2002)
23. Rozas, J. *et al*. DnaSP 6: DNA sequence polymorphism analysis of large data sets. *Mol. Biol. Evol*. **34**, 3299-3302 (2017).
24. Leigh, J. W., Bryant, D. & Nakagawa, S. POPART: full-feature software for haplotype network construction. *Methods Ecol. Evol*. **6**, 1110-1116 (2015).
25. Yi, H., Wang, J., Dong, S. & Kang, M. Genomic signatures of inbreeding and mutation load in tree ferns. *Plant J.* **120**, 1522–1535 (2024).
26. Ma, Y. *et al*. Demographic history and identification of threats revealed by population genomic analysis provide insights into conservation for an endangered maple. *Mol. Ecol.* **31**, 767-779 (2021).
27. Chen, J., Glemin, S. & Lascoux, M. Genetic diversity and the efficacy of purifying selection across plant and animal species. *Mol. Biol. Evol.* **34**, 1417–1428 (2017).
