# Supplementary material for: Decoding the genome of Brainea insignis reveals insights into fern evolution and conservation
Source: Nat Commun. 2025 Dec 30;17:1292. doi: 10.1038/s41467-025-68053-0 (PMC12868710; doi:10.1038/s41467-025-68053-0)
Supplement: Supplementary file 2 — Description of Addiational Supplemenatry Files [file 41467_2025_68053_MOESM2_ESM.pdf]

Description of Additional Supplementary Files

File Name: Supplementary Data 1
Description: Includes resequencing sample collection coordinates and elevations

File Name: Supplementary Data 2
Description: Contains worksheets that record the GO and KEGG annotations of loss-of-function (LOF) mutations.
