# Supplementary material for: Decoding the genome of Brainea insignis reveals insights into fern evolution and conservation
Source: Nat Commun. 2025 Dec 30;17:1292. doi: 10.1038/s41467-025-68053-0 (PMC12868710; doi:10.1038/s41467-025-68053-0)
Supplement: Supplementary file 5 — Reporting Summary [file 41467_2025_68053_MOESM5_ESM.pdf]

nature portfolio

Corresponding author(s): Yves Van de Peer, Ming Kang, Faguo Wang

Last updated by author(s): Dec 10, 2025

Reporting Summary

Nature Portfolio wishes to improve the reproducibility of the work that we publish. This form provides structure for consistency and transparency in reporting. For further information on Nature Portfolio policies, see our Editorial Policies and the Editorial Policy Checklist.

Statistics

For all statistical analyses, confirm that the following items are present in the figure legend, table legend, main text, or Methods section.

n/a	Confirmed	
☐	☒	The exact sample size (*n*) for each experimental group/condition, given as a discrete number and unit of measurement
☐	☒	A statement on whether measurements were taken from distinct samples or whether the same sample was measured repeatedly
☐	☒	The statistical test(s) used AND whether they are one- or two-sided<br>*Only common tests should be described solely by name; describe more complex techniques in the Methods section.*
☒	☐	A description of all covariates tested
☒	☐	A description of any assumptions or corrections, such as tests of normality and adjustment for multiple comparisons
☐	☒	A full description of the statistical parameters including central tendency (e.g. means) or other basic estimates (e.g. regression coefficient) AND variation (e.g. standard deviation) or associated estimates of uncertainty (e.g. confidence intervals)
☐	☒	For null hypothesis testing, the test statistic (e.g. *F*, *t*, *r*) with confidence intervals, effect sizes, degrees of freedom and *P* value noted<br>*Give P values as exact values whenever suitable.*
☒	☐	For Bayesian analysis, information on the choice of priors and Markov chain Monte Carlo settings
☐	☒	For hierarchical and complex designs, identification of the appropriate level for tests and full reporting of outcomes
☐	☒	Estimates of effect sizes (e.g. Cohen's *d*, Pearson's *r*), indicating how they were calculated

Our web collection on statistics for biologists contains articles on many of the points above.

Software and code

Policy information about availability of computer code

Data collection	Genome survey data was sequenced on the BGI DNBSEQ platform. Genome HiFi reads was generated by PacBio Sequel II/IIe platform. Whole genome re-sequencing was sequenced on the DNBSEQ-T7 platform.

Data analysis	For the analysis of genome survey, we used GCE (v.1.0.2). For the genome assembly and assessment, we used Hifiasm (v.0.19.5-r587), BWA (v.0.7.17-r1188), Juicer (v.1.6), 3D-DNA, BUSCO (v5), QUAST (v.5.2.0) and CRAQ (v.1.0.9).<br><br>For the genome annoation, we used RepeatMasker, MITE-Hunter, RepeatModeler (v.2.0.3) and DeepTE softwares. Protein-coding genes were predicted using ab initio, homology-based, and RNA-seq-assisted approaches (details in Supplementary Notes). Functional annotations were assigned via Blastp against Swiss-Port, InterProScan, Gene Ontology (GO) and Kyoto Encyclopedia of Genes and Genomes (KEGG).<br><br>For the comparative genomics analysis, we used fastp (v.0.22.0), WGDI (v0.6.1), wgd v2, LTR_Finder, LTRharvest, LTR_retriever, Orthofinder (v.2.5.5), MAFFT (v.7.520), trimAL (v.1.4.rev15), IQ-TREE (v.2.2.6), PAML (v.4.8), MEGA (v7), CAFE (v.4.2.1), KaKs_Calculator (v.3.0).<br><br>All the SNP variants datasets were generated by GATK (v.4.5.0.0).<br><br>For the population genetic related analysis, we used ADMIXTURE (v.1.3.0), PLINK (v.1.9), Picard (https://broadinstitute.github.io/picard/), PHYLIP (https://evolution.genetics.washington.edu/phylip.html), degenotate pipline (https://github.com/harvardinformatics/degenotate), WorldClim (https://worldclim.org/), Matplotlib (https://matplotlib.org/), PopART, pixy (v.1.2.10), VCFTools (v.0.1.16), PopLDdecay (v.3.42), BCFtools (v.1.14), PSMC, SMC++ (v.1.15.2), fastsimcoal2 (v.2.8), TreeMix (v.1.13), ANGSD (v.0.937), SnpEff (v.4.3t), SIFT-4G, SRplot, RaiSD (v.2.9), LEA (v.3.16.0) and vegan (v.2.6-8).

For manuscripts utilizing custom algorithms or software that are central to the research but not yet described in published literature, software must be made available to editors and reviewers. We strongly encourage code deposition in a community repository (e.g. GitHub). See the Nature Portfolio guidelines for submitting code & software for further information.

Data

Policy information about availability of data

All manuscripts must include a data availability statement. This statement should provide the following information, where applicable:

- Accession codes, unique identifiers, or web links for publicly available datasets
- A description of any restrictions on data availability
- For clinical datasets or third party data, please ensure that the statement adheres to our policy

Data supporting the findings of this work are available within the paper and its Supplementary Information files. The datasets and plant material generated and analyzed during the current study are available from the corresponding author upon request. All of the raw sequence datasets used in this study have been deposited at NCBI under the BioProject accession numbers PRJNA1203580. The genome assembly and annotation files are available at Figshare (DOI: 10.6084/m9.figshare.28229507). Source data are provided with this paper.

Research involving human participants, their data, or biological material

Policy information about studies with human participants or human data. See also policy information about sex, gender (identity/presentation), and sexual orientation and race, ethnicity and racism.

Reporting on sex and gender	n.a.
Reporting on race, ethnicity, or other socially relevant groupings	n.a.
Population characteristics	n.a.
Recruitment	n.a.
Ethics oversight	n.a.

Note that full information on the approval of the study protocol must also be provided in the manuscript.

Field-specific reporting

Please select the one below that is the best fit for your research. If you are not sure, read the appropriate sections before making your selection.

☐ Life sciences ☐ Behavioural & social sciences ☒ Ecological, evolutionary & environmental sciences

For a reference copy of the document with all sections, see nature.com/documents/nr-reporting-summary-flat.pdf

Ecological, evolutionary & environmental sciences study design

All studies must disclose on these points even when the disclosure is negative.

Study description	Here, we present a chromosome-level genome assembly (8.62 Gb) of Brainea insignis, an endangered cycad fern and the sole representative of its genus within the eupolypods II clade. The study involved generating a reference genome from a single individual and resequencing 94 individuals from 29 wild populations across Southeast Asia. The experimental units are individual plants, with each population represented by multiple independent samples. No experimental treatments were applied, and the design aimed to capture natural genetic variation across the species' distribution range. Comparative genomic analyses revealed unique evolutionary

	adaptations, including a whole-genome duplication event and genomic features associated with xylem development. Furthermore, population resequencing data allowed us to characterize the species' genetic structure, demographic history, and genomic evidence underlying its endangerment.
Research sample	A single adult Brainea insignis individual was collected from the South China Botanical Garden for reference genome sequencing. As a fern species, sex and age are not applicable. The specimen showed typical morphology and no visible pathogens. Fresh leaves were flash-frozen for DNA extraction, with no additional manipulations.For population analyses, 94 wild individuals from 29 natural populations across Southeast Asia were sampled to represent the species' geographic and environmental range. Individuals were spaced at least 100 m apart to avoid redundancy. Leaf tissues were collected under permits and dried in silica gel. Public datasets used for comparative analyses were obtained from PRJNA593361, PRJNA729743, PRJCA006485, and PRJNA1234874, and are cited in the manuscript.
Sampling strategy	The reference genome was generated from a single, morphologically representative B. insignisindividual. We performed population genomic sequencing on 94 individuals from 29 populations spanning Southeast Asia (mean of 3.2 individuals per site), a sample size that exceeds the typical minimum recommendation. Specimens were selected based on availability and to ensure broad geographical coverage across the species' range, balancing logistical constraints.
Data collection	Field sampling was conducted from 2022 to 2023 by trained botanists from the South China Botanical Garden (Faguo Wang Lab). Zengqiang Xia and Faguo Wang, both authors of this study, were responsible for collecting and recording all samples. Geolocation and elevation data were recorded using BioTracks (v3.02), and each specimen was immediately geotagged and preserved in silica gel or liquid nitrogen to ensure DNA integrity. Detailed protocols for HiFi, Hi-C, and resequencing data generation are provided in the corresponding Methods sections.
Timing and spatial scale	Field sampling was conducted from January 2022 to October 2023. Genomic sequencing data were sourced from individuals at the South China Botanical Garden, while population-level sequencing data spanned multiple locations across Southeast Asia. Specific collection information has been included in the Supplementary Data.
Data exclusions	No data were excluded from the reference genome assembly or population analyses.
Reproducibility	To ensure the reproducibility and robustness of our population genetic analyses, we implemented the following measures for key analytical steps: (1) For population structure inference using ADMIXTURE, we employed a cross-validation procedure to objectively identify the most supported number of genetic clusters (K-value). (2) For modeling historical population splits and gene flow with TreeMix, we independently ran the analysis multiple times with different random seeds to confirm the stability of the inferred tree topology and migration edges. (3) For phylogenetic reconstruction, we assessed branch support by performing 1,000 bootstrap replicates. (4) To ensure robustness in demographic inference, we rigorously evaluated a total of 10 demographic and 5 differentiation models. Each model was run with 100 independent replicates to assess convergence and stability. This extensive replication allowed us to confidently select the best-fitting scenario based on the lowest AIC and ΔLhood values, ensuring that our conclusions are not contingent on a single stochastic optimization. All attempts to generate the reference genome and resequencing data were successful. DNA extractions, library preparations, and sequencing runs were repeated as needed, and no failures occurred.
Randomization	Samples were initially grouped based on location,and were then classified into populations after investigating population structure between samples.
Blinding	Blinding was not performed because this study does not measure or evaluate an exposure

Did the study involve field work? ☐ Yes ☒ No

Reporting for specific materials, systems and methods

We require information from authors about some types of materials, experimental systems and methods used in many studies. Here, indicate whether each material, system or method listed is relevant to your study. If you are not sure if a list item applies to your research, read the appropriate section before selecting a response.

Materials & experimental systems

n/a	Involved in the study
☒	☐ Antibodies
☒	☐ Eukaryotic cell lines
☒	☐ Palaeontology and archaeology
☒	☐ Animals and other organisms
☒	☐ Clinical data
☒	☐ Dual use research of concern
☐	☒ Plants

Methods

n/a	Involved in the study
☒	☐ ChIP-seq
☐	☒ Flow cytometry
☒	☐ MRI-based neuroimaging

Dual use research of concern

Policy information about dual use research of concern

Hazards

Could the accidental, deliberate or reckless misuse of agents or technologies generated in the work, or the application of information presented in the manuscript, pose a threat to:

No	Yes	
☒	☐	Public health
☒	☐	National security
☒	☐	Crops and/or livestock
☒	☐	Ecosystems
☒	☐	Any other significant area

Experiments of concern

Does the work involve any of these experiments of concern:

No	Yes	
☒	☐	Demonstrate how to render a vaccine ineffective
☒	☐	Confer resistance to therapeutically useful antibiotics or antiviral agents
☒	☐	Enhance the virulence of a pathogen or render a nonpathogen virulent
☒	☐	Increase transmissibility of a pathogen
☒	☐	Alter the host range of a pathogen
☒	☐	Enable evasion of diagnostic/detection modalities
☒	☐	Enable the weaponization of a biological agent or toxin
☒	☐	Any other potentially harmful combination of experiments and agents

Plants

Seed stocks	Genomic sequencing samples were obtained from the South China Botanical Garden, Chinese Academy of Sciences (CAS). An additional 94 wild accessions were collected from natural populations, with detailed geographic coordinates provided in Supplementary Data.
Novel plant genotypes	n.a.
Authentication	Species identity of wild samples was confirmed by population genetics analysis.

Flow Cytometry

Plots

Confirm that:

☒ The axis labels state the marker and fluorochrome used (e.g. CD4-FITC).

☒ The axis scales are clearly visible. Include numbers along axes only for bottom left plot of group (a 'group' is an analysis of identical markers).

☐ All plots are contour plots with outliers or pseudocolor plots.

☐ A numerical value for number of cells or percentage (with statistics) is provided.

Methodology

Sample preparation	For flow cytometry analysis, fresh leaves from four test samples were placed in 0.8 mL of pre-chilled MGb lysis buffer, finely chopped, and incubated on ice for 10 minutes. The resulting suspension was filtered through a 40-micron mesh to isolate nuclei. Camellia sinensis var. assamica served as the internal standard. Nuclear suspensions from the test samples and the standard were mixed, stained, and analyzed on a BD FACScalibur flow cytometer with 488 nm excitation for detecting propidium iodide (PI) fluorescence.
Instrument	BD FACScalibur
Software	Using Modifit v.3.0 (Verity Software House, Topsham, ME, USA), we compared the PI-DNA fluorescence peaks between the test samples and the reference plant, and calculated the DNA content ratio to determine the C-value of the test samples.
Cell population abundance	Regarding the determination of genome size and C-value, this typically involves comparing fluorescence intensities to estimate DNA content, rather than directly measuring the abundance of cell populations.
Gating strategy	In this study, the gating strategy was designed to select and analyze cells based on their fluorescence intensity for accurate genome size (C-value) determination rather than isolating specific cell populations. The primary focus was on establishing a clear distinction between the internal standard and the sample cells by comparing their fluorescence signals.

☒ Tick this box to confirm that a figure exemplifying the gating strategy is provided in the Supplementary Information.
